# Circular RNA FOXP1 promotes tumor progression and Warburg effect in gallbladder cancer by regulating PKLR expression

**DOI:** 10.1186/s12943-019-1078-z

**Published:** 2019-10-17

**Authors:** Shouhua Wang, Yongjie Zhang, Qiang Cai, Mingzhe Ma, Long Yang Jin, Mingzhe Weng, Di Zhou, Zhaohui Tang, Jian Dong Wang, Zhiwei Quan

**Affiliations:** 1 Department of General Surgery, Xinhua Hospital, Shanghai Jiao Tong University School of Medicine, No.1665 Kong jiang Road, Yangpu District, Shanghai, 200000 China; 2 Department of Molecular Oncology & Biliary Tract Surgery, Eastern Hepatobiliary Surgery Hospital, National Center of Liver Cancer, Second Military Medical University, Shanghai, China; 3 Department of Gastric Cancer and Soft Tissue Sarcoma, Fudan University Shanghai Cancer Center, Shanghai, China

**Keywords:** Gallbladder cancer, circFOXP1, PTBP1, PKLR, miR-370

## Abstract

**Background:**

Circular RNAs (circRNAs) have recently been identified as potential functional modulators of the cellular physiology processes. The study aims to uncover the potential clinical value and driving molecular mechanisms of circRNAs in gallbladder cancer (GBC).

**Patients and methods:**

We performed RNA sequencing from four GBC and paired adjacent normal tissues to analyze the circRNA candidates. Quantitative real-time polymerase chain reaction (QRT-PCR) was used to measure the circFOXP1 expression from 40 patient tissue samples. Short hairpin RNA mediated knockdown or exogenous expression of circFOXP1 combined with in vitro and in vivo assays were performed to prove the functional significance of circFOXP1. Double luciferase reporter, RNA immunoprecipitation (RIP) and RNA pull-down assays were also performed.

**Results:**

By performing RNA sequencing from GBC and paired adjacent normal tissues to analyze the circRNA candidates, we identified that circFOXP1 (hsa_circ_0008234) expression was significantly upregulated in GBC tissues and positively associated with lymph node metastasis, advanced TNM stage and poor prognosis in patients. Short hairpin RNA mediated knockdown or exogenous expression of circFOXP1 combined with in vitro assays demonstrated that circFOXP1 has pleiotropic effects, including promotion of cell proliferation, migration, invasion, and inhibition of cell apoptosis in GBC. In vivo, circFOXP1 promoted tumor growth. Mechanistically, double luciferase reporter, RNA immunoprecipitation (RIP) and biotin-labeled RNA pull-down assays clarified that circFOXP1 interacted with PTBP1 that could bind to the 3’UTR region and coding region (CDS) of enzyme pyruvate kinase, liver and RBC (PKLR) mRNA (UCUU binding bites) to protect PKLR mRNA from decay. Additionally, circFOXP1 acted as the sponge of miR-370 to regulate PKLR, resulting in promoting Warburg effect in GBC progression.

**Conclusions:**

These results demonstrated that circFOXP1 serve as a prognostic biomarker and critical regulator in GBC progression and Warburg effect, suggesting a potential target for GBC treatment.

## Background

Gallbladder cancer (GBC) is the most common and leading cause of cancer-associated mortality among biliary tract carcinomas worldwide. Complete surgical resection is the only potentially curative choice for GBC, but due to lack of specific signs, symptoms or reliably sensitive disease markers, many patients are diagnosed at advanced stages [1, 2]. Conventional chemotherapy, radiotherapy, and molecular targeted therapy provide palliative relief for GBC patients; however, in the past few decades, there has been no definitive improvement in overall survival (OS) [3–5]. Therefore, improving molecular understanding of the underlying pathogenesis in GBC and investigating mechanism-based therapeutic strategies for patients is essential.

Circular RNAs (circRNAs), covalently closed RNAs formed from both exonic and intronic sequences, are highly stable RNA molecules [6, 7]. CircRNAs exert certain functions, including as intermediates in RNA alternative splicing (AS), as regulators of transcription in cis, and as miRNA sponges [8]. Some of circRNAs are identified as potential functional modulators of cellular physiology processes involved in cell proliferation, differentiation, apoptosis and tumor progression [9, 10]. For example, CiRS-7 (also termed CDR1as) harbors more than 70 conventional miR-7 binding sites and is involved in cancer-related pathways [11]. CircHIPK3 but not HIPK3 mRNA regulates human cell growth in hepatocellular carcinoma [12]. Ectopic expression of circ-foxo3 repressed cell cycle progression by binding to cyclin-dependent kinase 2 (CDK2) and cyclin-dependent kinase inhibitor 1 (or p21) [13].

The Warburg effect (or aerobic glycolysis) is a well-characterized metabolic alteration that involved in cancer phenomenon including rapid cell proliferation, invasion, and migration in tumors [14]. enzyme pyruvate kinase, liver and RBC (PKLR) is identified as a pivotal regulator of glycolytic reprogramming and affect tumor cells. Nie et al. showed that mineralocorticoid receptor (MR) inhibits the Warburg effect and cancer progression via the miR-338-3p-PKLR axis in hepatocellular carcinoma [15]. Yang et al. demonstrated the NQO1/PKLR axis promotes lymph node metastasis and breast cancer progression by activating the AMPK and AKT/mTOR signaling pathway and consequently induced glycolytic reprogramming [16]. The association of between circRNAs and Warburg effect in gallbladder cancer remains unknown.

Here, we identified a circular FOXP1 RNA (circFOXP1, hsa_circ_0008234), derived from the exon region of the FOXP1 gene [17, 18], which is significantly upregulated in GBC tissues. CircFOXP1 interacted with PTBP1 or sponged miR-370, which promoted tumor progression and the Warburg effect in GBC by directly targeting the PKLR. Our results indicate that circFOXP1 may be a novel potential target for GBC treatment.

## Methods

### RNA-sequencing analysis

A detailed description of RNA-sequencing analysis was provided in Additional file 1: Supplementary Materials and Methods section. The GEO Accession number is GSE100363.

### Patient tissue samples

Forty human GBC and adjacent normal tissue specimens were collected from patients who underwent radical resection at Xinhua Hospital between March 2009 and January 2013. The eleven patients were male and twenty-nine were female. Ages ranged from 35 to 82 years (the mean value = 54.85 years). Each tissue sample was snap-frozen in liquid nitrogen for further analysis. All of patients in this study belonged to the same ethnic group. The patients were selected according to the criteria: (1) All clinicopathological diagnoses were confirmed by two pathologists. (2) None of the patients received any treatments before surgery. (3) None of the patients received radiotherapy or chemotherapy during follow-up period. (4) availability of complete follow-up data and not lost follow-up. (5) no death in the perioperative period. (6) no history of other synchronous malignancies. (7) For patients undergoing radical resection of gallbladder cancer. The study procedure was approved by the Human Ethics Committee of Xinhua Hospital. All patients signed consent forms. Follow-ups after surgery were performed according to patient survival time until March 8, 2016. The clinicopathological data were shown in Table 1.

**Table 1:** Correlation between circFOXP1 expression and clinicopathological characteristics in 40 cases GBC patients

		CircFOXP1 expression		*P*-value
Clinicopathological characteristics	The number of patients	Lower (*n* = 20)	Higher (*n* = 20)	
Age				
≤ 60	25	10	15	0.102
> 60	15	10	5	
Gender				
Male	11	6	5	0.723
Female	29	14	15	
Tumor size				
< 5 cm	16	11	5	0.053
≥ 5 cm	24	9	15	
Histological grade				
well and moderately	21	10	11	0.752
Poorly and others	19	10	9	
Lymph node metastasis				
N0	18	13	5	0.011*
N1/2	22	7	15	
TNM stage				0.027*
I-II	19	13	6	
III-IV	21	7	14	
Adjacent organ invasion				0.110
No	17	11	6	
Yes	23	9	14	

*P < 0.05.*TNM* tumor-node-metastasis

### Cell culture

Human GBC cell lines NOZ, GBC-SD, EHGB-1, SGC-996 and OCUG-1 and the human intrahepatic biliary epithelial cell line H69 and another normal biliary epithelia cell line HIBEC were used in the present study. GBC-SD and OCUG-1 cell lines were purchased from Cell Bank of the Chinese Academy of Science (Shanghai, China). The NOZ cell line was purchased from the Health Science Research Resources Bank (Osaka, Japan). EHGB-1 and SGC-996 cells were a generous gift from Eastern Hepatobiliary Surgical Hospital and Institute, The Second Military University, Shanghai, China. The cells were cultured in Dulbecco’s modified Eagle’s medium (Gibco BRL, Grand Island, NY, USA); the cell media contained 10% fetal bovine serum (FBS, HyClone, Invitrogen). Cells were maintained in a humidified incubator at 37 °C in the presence of 5% CO_2_.

### RNAi, plasmid construction and cell transfection

Cells were transfected using lipofectamine 2000 according to the manufacturer’s instructions. Additional information about the performed experiments can be found in the Additional file 1: Supplementary Materials and Methods. The sequences of all oligonucleotide used in the study were shown in Additional file 2: Table S1.

### Cell proliferation, migration and invasion assays and cell cycle and cell apoptosis analysis

Cell proliferation was assessed using Cell Counting Kit 8 (Dojindo, Japan); cell migration and invasion was assessed with transwell assays. Cell cycle distribution and cell apoptosis rate analysis was analyzed using flow cytometry. Detailed descriptions of experiments can be found in Additional file 1: Supplementary Materials and Methods.

### RNA isolation, quantitative real-time PCR (qRT-PCR) and western blot analysis

RNA isolation, qRT-PCR and western blot analysis were performed as described previously [19]. Detailed descriptions of experiments can be found in Additional file 1: Supplementary Materials and Methods. The sequences of all primers used in the study were shown in Additional file 2: Table S1.

### Xenograft mouse model

NOZ cells (1 × 10^6^) stably expressing sh-NC, sh-circFOXP1–1, sh-circFOXP1–2 or GBC-SD cells (1 × 10^6^) stably expressing pLCDH-vector or pLCDH-circFOXP1 were subcutaneously injected into either side of the flank area of 3-week-old male nude mice (*n* = 5 mice per group). Tumor volumes were measured (0.5 × length × width^2^) and tumor weights were evaluated in mice on a weekly basis. After 4 weeks, mice were sacrificed, and the tumors were excised. The study protocol was approved by the Animal Care and Use committee of Xinhua Hospital (approval ID: 2014041). All animal experiments were performed in the animal laboratory center at Xinhua Hospital and conformed to the Guide for the Care and Use of Laboratory Animals published by the US National Institutes of Health (NIH publication number 85–23, revised 1996).

### RNA pull-down assay

RNA pull-down assays were performed as described previously [20, 21]. Briefly, cells extract (2 μg) was mixed with biotinylated RNA (100 pmol). Washed streptavidin agarose beads (100 ml) were added to each binding reaction and further incubated at room temperature for 1 h. Beads were washed briefly three times and boiled in SDS buffer, and the retrieved protein was determined by western blot analysis.

### RNA immunoprecipitation (RIP) assay

RIP assays were performed using an EZ-Magna RIP™ RNA-Binding Protein Immunoprecipitation Kit (Millipore, Billerica, MA, USA) according to the manufacturer’s instructions. Cells at approximately 90% confluence was lysed using complete RIP lysis buffer containing RNase Inhibitor (Millipore) and protease inhibitor and then 100 μl of whole cell extract was incubated with RIP buffer containing magnetic beads conjugated to specific antibodies. The negative control was normal mouse anti-IgG antibody (Cell Signaling Technology, USA), and the positive control was anti-SNRNP70 antibody (Millipore, USA).

### Statistical analysis

All experiments were independently repeated at least three times. Statistical analyses were performed using SPSS 20.0 (SPSS, Chicago, IL, USA). The data are expressed as the mean ± standard error of the mean (SEM). OS was estimated using the Kaplan-Meier method and a log-rank test. The difference between groups was analyzed using Student’s t-test. Differences were statistically significant at *P* < 0.05.

## Results

### Expression of circFOXP1 is significantly upregulated in GBC tissues and cells

**Fig. 1:**
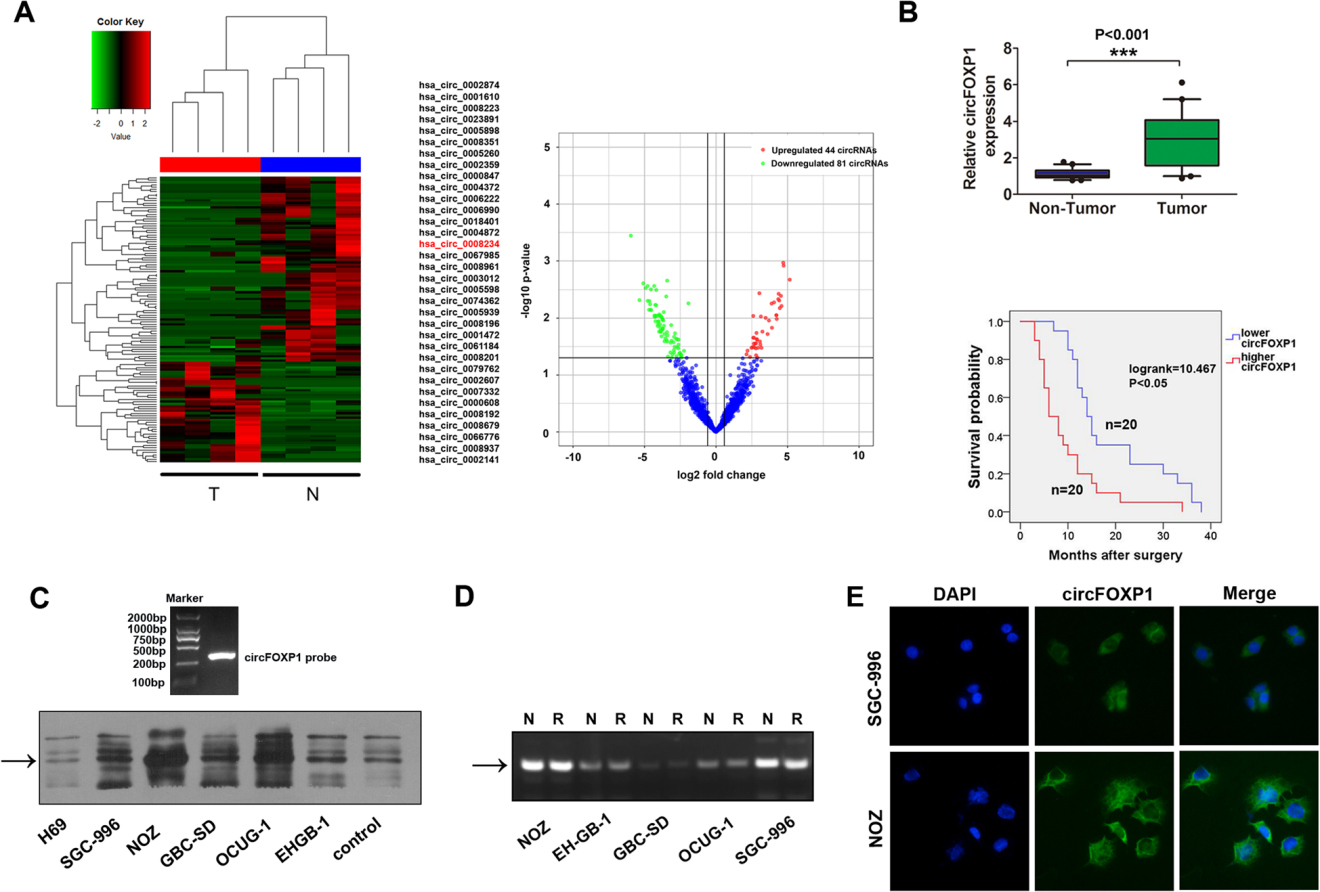
circFOXP1 is upregulated in GBC tissues. **a** The heat map showed that the upregulated 44 circRNAs and downregulated 81 circRNAs in GBC tissues when compared with adjacent non tumor tissues analyzed by RNA sequencing. The 34 of the upregulated 44 circRNAs were overlapped in database obtained from circBase. **b** Top, circFOXP1 expression levels were evaluated with qRT-PCR in tissues from 40 cases of GBC compared with adjacent normal tissues. The expression of circFOXP1 was normalized to GADPH. Significant differences between groups were analyzed with a paired samples *t-*test, ***P < 0.001. Low, Kaplan-Meier analysis and log-rank tests were performed to analyze the association between the expression of circFOXP1 and OS time of GBC patients. CircFOXP1 expression levels were classified as higher expression (circFOXP1 expression rate > median expression rate) and lower expression (circFOXP1 expression rate < median expression rate). **c** RNAs were isolated from five GBC cell lines and the H69 cell line and subjected to a northern blot analysis using a probe specific for circFOXP1 (top). Arrow, circFOXP1 expression was shown. **d** Standard PCR was performed to detect the circFOXP1 from control RNA or digested RNAs using RNase R exonuclease in five GBC cell lines. Arrow, circFOXP1 expression was shown. **e** RNA fluorescence in situ hybridization (FISH) was performed for circFOXP1. Nuclei were stained with 4, 6-diamidino-2-phenylindole (DAPI); the original magnification was 400×

To explore circRNAs expression profile in GBC, we performed RNA sequencing analyses of ribosomal RNA-depleted total RNA from four pairs of GBC tissues and adjacent normal tissues. The cluster heat map demonstrated the differentially expressed circRNAs over 3.5-fold change (Fig. 1a, left). The scatter plots showed that upregulated 44 circRNAs and downregulated 81 circRNAs in GBC tissues compared with adjacent normal tissues (Fig. 1a, right). Compared with previously reported database obtained from circBase [18], 34 of the top 44 upregulated circRNAs were overlapped circRNAs and were listed in Fig. 1a, left. Several circRNAs including circXPO1 (chr2_61710091_61717911_-, hsa_circ_0002607), circFOXP1 (chr3_71090478_71102924_-,hsa_circ_0008234),circMAPK1(chr22_22153300_22162135_-, hsa_circ_0004872), circSENP1 (chr12_48477373_48491907_-, hsa_circ_0006222) and circSMAD2 (chr18_45391429_45423180_-, hsa_circ_0000847) were chosen for further investigation because their parental genes are involved in cell proliferation, invasion and cell cycle regulation in some tumors [22–26]. We examined these investigated circRNA candidates in GBC tissues and paired normal tissue samples from 40 patients. The results showed that expression of circFOXP1, circMAPK1 and circSAMD2 were upregulated in GBC tissues (Fig. 1b, top and Additional file 3: Figure S1A). GBC patients were classified into two groups: high-circFOXP1 group (*n* = 20, circFOXP1 expression ratio ≥ median ratio) and low-circFOXP1 group (*n* = 20, circFOXP1 expression ratio < median ratio). Kaplan-Meier analysis and log rank tests indicated that higher circFOXP1 expression levels were positively correlated with a shorter OS in patients (Fig. 1b, low), while circSMAD2 and circMAPK1 expression showed no statistical significance with OS in patients (Additional file 3: Figure S1B). CircFOXP1 expression was upregulated in several tumors compared with normal tissues through RNA sequencing data in a previous study [12]. However, the clinical significance and functional role of circFOXP1 remains unknown.

As seen in Table 1, the statistical analysis revealed that high circFOXP1 expression levels were strongly correlated with lymph node metastasis (*P* = 0.011) and advanced TNM stage (III-IV) (*P* = 0.027) in GBC patients, but no correlation was found with the other factors (Table 1). Univariate and multivariate Cox analysis revealed that lymph node metastasis (HR = 3.749, 95% CI: 1.578–8.905, *P* < 0.05) and high circFOXP1 expression (HR = 3.658, 95% CI: 1.587–8.431, P < 0.05) were independent risk factors for the OS of GBC patients (Table 2). As shown in Additional file 4: Figure S2A, the transcript levels of circFOXP1 were also significantly higher in several GBC cells than in the bile duct epithelial cell line H69 or another normal biliary epithelia cell line HIBEC. RNA was isolated and RT-PCR with Sanger sequencing confirmed the circular form in GBC cells (Additional file 4: Figure S2B). RNA isolated from the above cell lines was subjected to northern blot using a probe specific for circFOXP1, confirming that circFOXP1 was present in these cells (Fig. 1c). In addition, we confirmed that circFOXP1 was resistant to RNase R after digestion of RNA using RNase R exonuclease (Fig. 1d and Additional file 4: Figure S2C), which was consistent with a previous study [27], the circHIPK3 was used as the control, which was reported as a circRNA in previous study [12]. Using fluorescence in situ hybridization (FISH) and qRT-PCR analysis, we also noted that circFOXP1 localized in the cytoplasm and nucleus, but was predominately enriched in the cytoplasm in GBC cells (Fig. 1e and Additional file 4: Figure S2D). Thus, the above results indicated that circFOXP1 may serve as a prognostic biomarker for GBC.

**Table 2:** Univariate and multivariate Cox analysis of the overall survival (OS) in 40 GBC patients

Factors	Univariate Cox analysis			Multivariate Cox analysis		
	HR	95% CI	*P*-value	HR	95% CI	*P*-value
Age	1.005	0.974–1.037	0.754			
Gender	0.796	0.390–1.624	0.530			
Tumor size	0.917	0.484–1.738	0.790			
Histological grade	1.426	0.755–2.693	0.274			
Lymph node metastasis	2.938	1.476–5.848	0.002*	3.749	1.578–8.905	0.003*
TNM stage	2.105	1.064–4.164	0.033*	1.123	0.372–2.032	0.747
Adjacent organ invasion	1.985	1.003–3.930	0.049*	0.968	0.447–2.317	0.896
Higher circFOXP1	2.762	1.422–5.362	0.003*	3.658	1.587–8.431	0.002*

*P < 0.05, *HR* Hazard Ratio, *CI* Confidence intervals

### Overexpression of circFOXP1 promotes GBC cell growth in vitro and in vivo

**Fig. 2:**
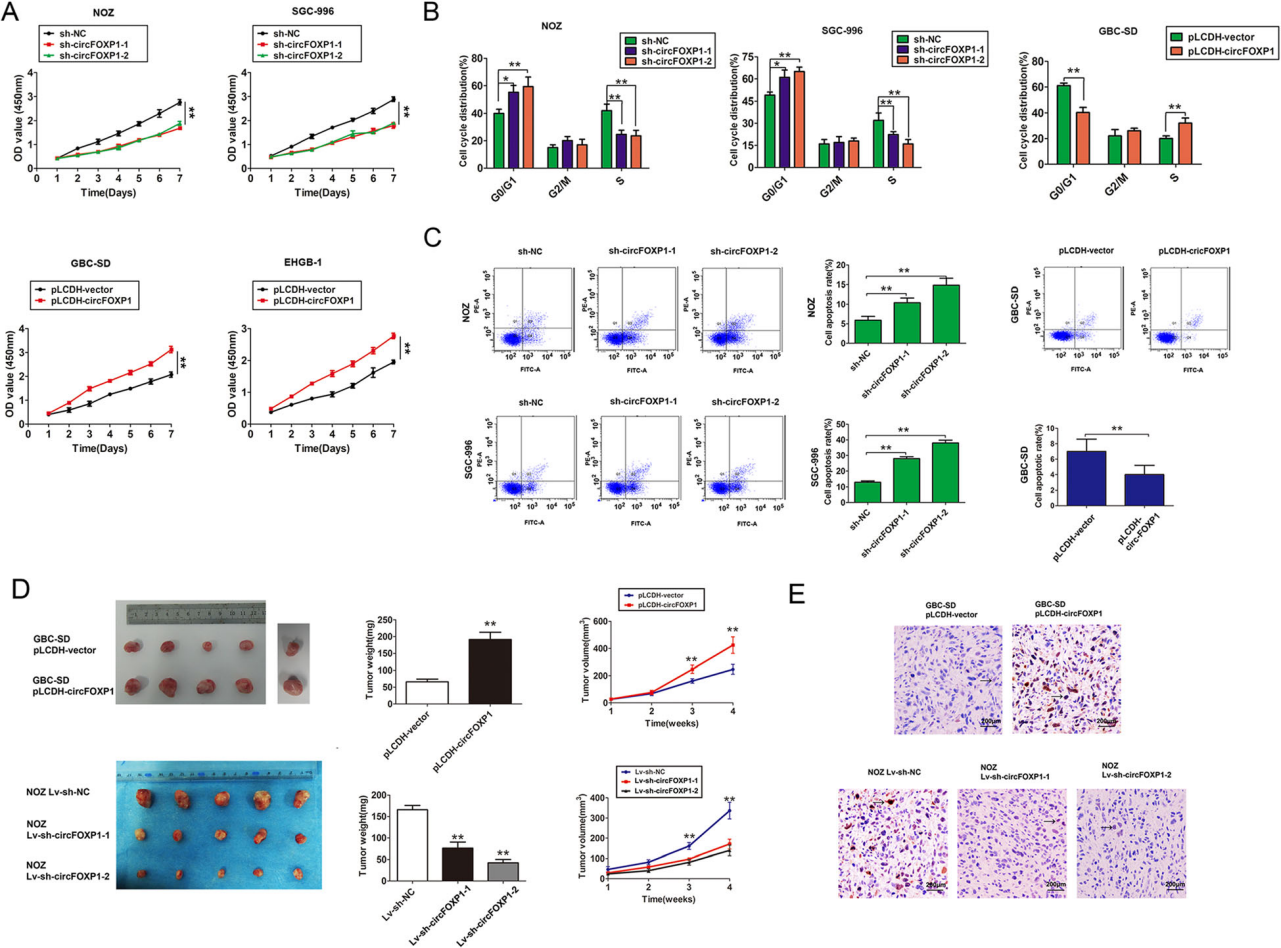
circFOXP1 promotes cell proliferation in vitro and in vivo. **a** Cell proliferation capacity was evaluated with CCK8 assays. Briefly, 2000 cells/well were plated in triplicate and cell proliferation was detected at days 1, 2, 3, 4, 5, 6 and 7 days after transfection of NOZ and SGC-996 cells (top) with sh-NC, sh-circFOXP1–1 or sh-circFOXP1–2 or of GBC-SD and EHGB-1 cells (low) with pLCDH-vector and pLCDH-circFOXP1. The experiment was repeated three times, **P < 0.01. **b** Data are presented as the percentage cell phase distribution including G0/G1, S and G2/M phases after transfection of NOZ and SGC-996 cells with sh-NC, sh-circFOXP1–1 or sh-circFOXP1–2 or GBC-SD cells with pLCDH-vector and pLCDH-circFOXP1, *P < 0.05; **P < 0.01. **c** The data are presented as cell apoptosis rates after transfection of NOZ and SGC-996 cells (left) with sh-NC, sh-circFOXP1–1 or sh-circFOXP1–2 or GBC-SD cells (right) with pLCDH-vector and pLCDH-circFOXP1. **P < 0.01. **d** Tumor weight and volume were detected to monitor tumor growth in subcutaneous implantation mouse models; mice were implanted with GBC-SD cells (top) transfected with pLCDH-vector or pLCDH-circFOXP1 and NOZ cells (low) transfected with lv-sh-NC, lv-sh-circFOXP1–1 or lv-sh-circFOXP1–2. **e** Immunohistochemical staining of Ki67 expression was shown in tumor tissues, as indicated by the number of Ki67-positive cells with GBC-SD cells (top) transfected with pLCDH-vector or pLCDH-circFOXP1 and NOZ cells (low) transfected with lv-sh-NC, lv-sh-circFOXP1–1 or lv-sh-circFOXP1–2, (original magnification, 200×)

To further explore the biological significance of circFOXP1 in GBC progression, gain-and loss-of-function studies were performed. Based on endogenous expression of circFOXP1 in several GBC cell lines, we developed NOZ and SGC-996 cells with circFOXP1 stably silenced by shRNA-circFOXP1 and EHGB1 or GBC-SD cells with circFOXP1 stably overexpressed using pLCDH-circFOXP1. Meanwhile, to confirm the specificity of circFOXP1 silencing or overexpression, we showed that the cells transfection had no effects on mRNA expression of FOXP1 in GBC cells. In addition, circFOXP1 was also overexpressed after digestion of RNA using RNase R by using pLCDH-circFOXP1 (Additional file 4: Figure S2E-2F). By performing CCK8 assays and flow cytometry analysis in vitro, we observed that knockdown of endogenous circFOXP1 significantly suppressed cell proliferation capacity, G1-S arrest, and increased the cell apoptosis rate in NOZ and SGC-996 cells, but upregulated expression of circFOXP1 dramatically promoted the cell proliferation capacity, beyond the G1-S transition, and reduced the cell apoptosis rate in GBC-SD cells (Fig. 2a-c). Consistent with the decreased cell proliferation capacity, NOZ and SGC-996 cells exhibited reduced expression levels of proliferating cell nuclear antigen (PCNA), MMP9 and AKT, but increased Caspase-3 expression after knockdown of endogenous circFOXP1, and conversely, upregulated expression of circFOXP1 had the opposite effects in GBC cells (Additional file 5: Figure S3A). As shown in Additional file 5: Figure S3B-3C, migration and invasion capacities were impaired in NOZ and SGC-996 cells after knockdown of endogenous circFOXP1, but were dramatically promoted in GBC-SD cells after circFOXP1 overexpression.

To evaluate the biological function of circFOXP1 in vivo, a xenograft tumor model was constructed by inoculating different clones of NOZ and GBC-SD cells subcutaneously into nude mice. The results confirmed that the mean tumor volumes and weights were larger and tumor growth was rapid in the upregulated expression of circFOXP1 group, compared with the control group. However, the tumor growth was inhibited, as shown by decreasing mean volumes and weights, in the knockdown of endogenous circFOXP1 group compared with the control group (Fig. 2d). Immunohistochemical staining of tumor tissues revealed an increased proportion of proliferating cells (Ki67+) in the upregulated expression of circFOXP1 group compared with the control group. In contrast, knockdown of endogenous circFOXP1 led to reduced expression of Ki67 compared with the control group (Fig. 2e). These results indicated that circFOXP1 promoted GBC growth in vitro and in vivo.

### Upregulation of circFOXP1 promotes the Warburg effect in GBC cells

**Fig. 3:**
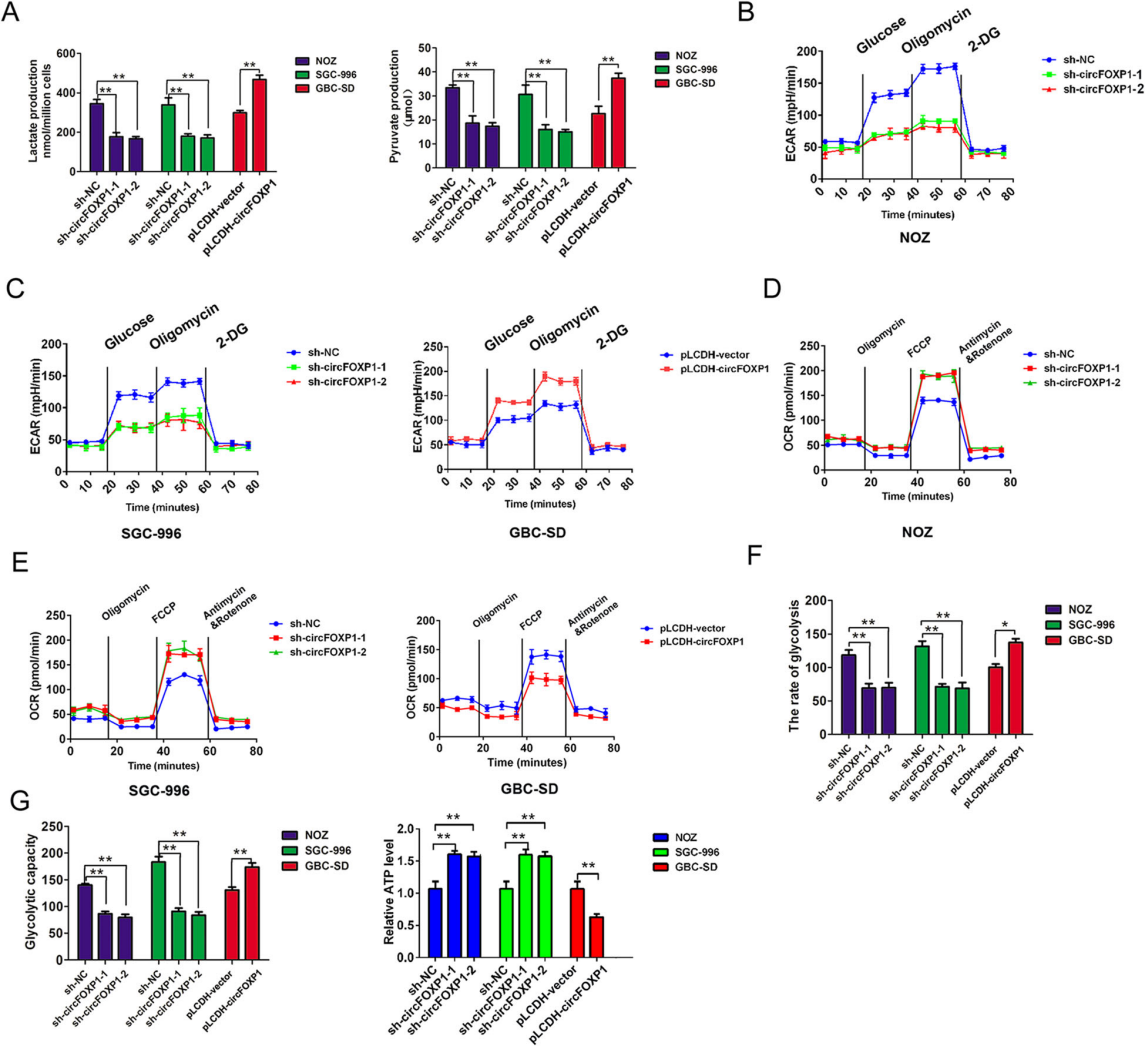
circFOXP1 promotes the Warburg effect in GBC cells. **a** Alterations in lactate production (left) and pyruvate production (right) levels were analyzed after transfection of NOZ and SGC-996 cells with sh-NC, sh-circFOXP1–1 or sh-circFOXP1–2 or GBC-SD cells with pLCDH-vector and pLCDH-circFOXP1, **P < 0.01. **b, c** The extracellular acidification rate was analyzed after transfection of NOZ and SGC-996 cells with sh-NC, sh-circFOXP1–1 or sh-circFOXP1–2 or GBC-SD cells with pLCDH-vector and pLCDH-circFOXP1. **d**, **e** The oxygen consumption rate was analyzed after transfection of NOZ and SGC-996 cells with sh-NC, sh-circFOXP1–1 or sh-circFOXP1–2 or GBC-SD cells with pLCDH-vector and pLCDH-circFOXP1. **f**, **g** The glycolysis rate, glycolysis capacity and ATP level was detected after transfection of NOZ and SGC-996 cells with sh-NC, sh-circFOXP1–1 or sh-circFOXP1–2 or GBC-SD cells with pLCDH-vector and pLCDH-circFOXP1. The extracellular acidification rate after glucose treatment indicates the glycolysis rate. The extracellular acidification rate after oligomycin treatment indicates the glycolysis capacity. Abbreviations: 2-DG, 2-deoxy-d-glucose; ECAR, extracellular acidification rate, **P < 0.01

The Warburg effect, characterized by abnormal metabolic phenomena that enhance glycolysis and reduce oxidative phosphorylation, induces significant differences between cancer cells and normal cells and affects tumor progression [28]. Based on the above findings, we investigated whether the expression of circFOXP1 affected the Warburg effect by measuring the levels of pyruvate production and lactate production and the glycolytic rate in GBC cells. The statistical analysis revealed a dramatically lower level of lactate production and pyruvate production after circFOXP1 knockdown in NOZ and SGC-996 cells, conversely, were significantly higher after circFOXP1 overexpression in GBC-SD cells (Fig. 3a). Furthermore, glycolysis and extracellular acidification rates (ECAR) were analyzed using an XF24 Extracellular Flux analyzer (Seahorse), The results confirmed that circFOXP1 knockdown reduced the ECAR in NOZ and SGC-996 cells, while upregulated circFOXP1 expression increased ECAR in GBC-SD cells (Fig. 3b-c). we also measured oxygen consumption rate (OCR, a marker of OXPHOS) and found that knockdown of circFOXP1 enhanced OCR ability in NOZ and SGC-996 cells, in contrast, upregulated circFOXP1 expression reduced OCR ability in GBC-SD cells (Fig. 3d-e). The results confirmed that circFOXP1 knockdown impaired the glycolysis rate and glycolytic capacity in NOZ and SGC-996 cells. On the other hand, upregulated circFOXP1 expression significantly enhanced the rate of glycolysis and glycolytic capacity in GBC-SD cells (Fig. 3f-g). In addition, knockdown of circFOXP1 in NOZ and SGC-996 cells also elevated the formation of ATP produced by OXPHOS, but upregulated circFOXP1 has an decreased formation of ATP in GBC-SD cells (Fig. 3g). These results indicated that upregulation of circFOXP1 promoted the Warburg effect in GBC cells.

### The RNA-binding protein PTBP1 binds to circFOXP1 in GBC cells

**Fig. 4:**
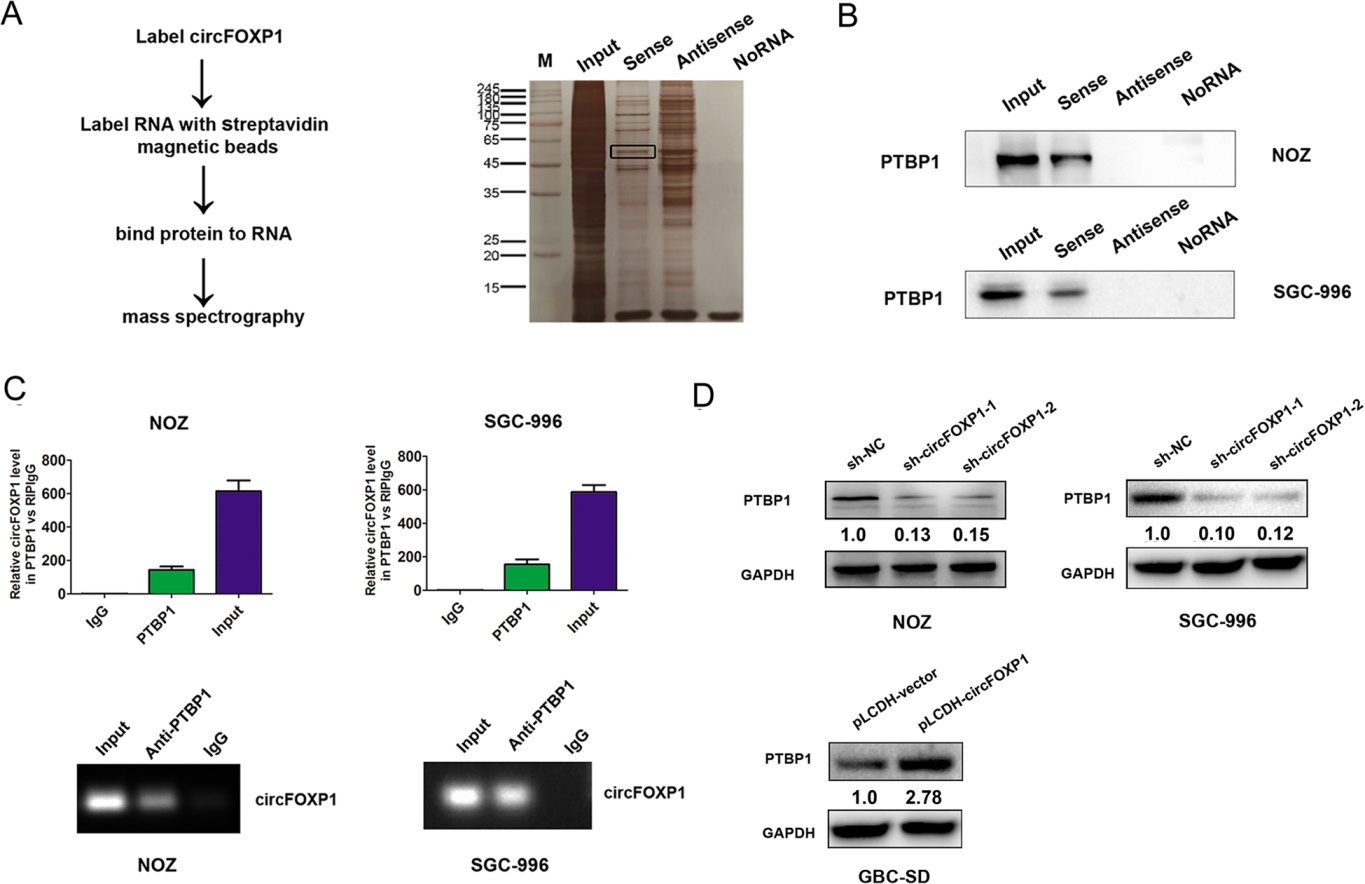
circFOXP1 interacts with PTBP1 in GBC cells. **a** A RNA pull-down assay was performed (left) and the RNA-related proteins were determined with SDS-polyacrylamide gel electrophoresis (SDS/PAGE) and silver staining (right). **b** PTBP1 was pulled down by a circFOXP1 sense RNA probe but not by an antisense RNA probe or no RNA in NOZ and SGC-996 cells. Western blot analysis was performed to detect the specific association of PTBP1 and circFOXP1 (*n* = 3). **c** RIP assays with qRT-PCR (top) or RT-PCR (low) showed that circFOXP1 was pulled down by an anti-PTBP1 antibody in NOZ and SGC-996 cells (*n* = 3). **d** The relative protein expression of PTBP1 after transfected with sh-NC, sh-circFOXP1–1 or sh-circFOXP1–2 in NOZ and SGC-996 cells and transfected with pLCDH-vector or pLCDH-circFOXP1 in GBC-SD cells were determined by western blot analysis (*n* = 3). All data are shown as mean ± S.E.M., n = 3 or more, **P < 0.01

**Fig. 5:**
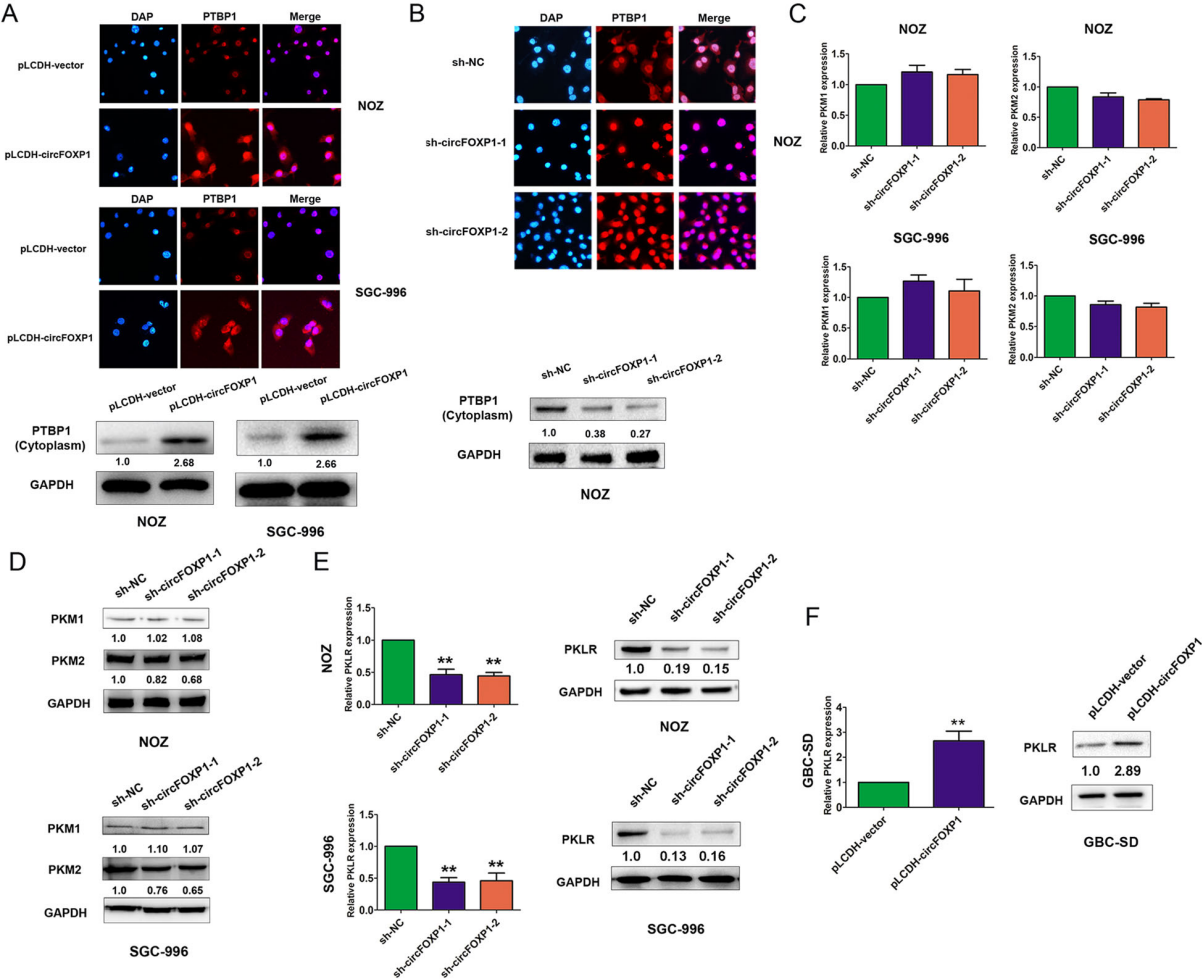
Effects of circFOXP1 on PTBP1, PKM1, PKM2 and PKLR expression in GBC cells. **a**, **b** The relative protein expression of PTBP1 after transfected with pLCDH-vector and pLCDH-circFOXP1 in NOZ and SGC-996 cells or sh-NC, sh-circFOXP1–1 and sh-circFOXP1–2 in NOZ cell were determined by immunofluorescence assays, original magnification, 200×. **c** The mRNA expression of PKM1 and PKM2 were determined with qRT-PCR after transfection of NOZ (top) and SGC-996 cells (low) with sh-NC, sh-circFOXP1–1 or sh-circFOXP1–2. **d** The relative protein expression of PKM1 or PKM2 after transfected with sh-NC, sh-circFOXP1–1 or sh-circFOXP1–2 in NOZ and SGC-996 cells was determined by western blot analysis (*n* = 3). **e**, **f** The mRNA and protein expression of PKLR were determined with qRT-PCR and western blot analysis after transfection of NOZ and SGC-996 cells with sh-NC, sh-circFOXP1–1 or sh-circFOXP1–2 or GBC-SD cells with pLCDH-vector and pLCDH-circFOXP1. All data are shown as mean ± S.E.M., *n* = 3 or more, **P < 0.01

We further investigated the mechanisms by which circFOXP1 promoted the Warburg effect in GBC cells. Recent evidence has indicated that circRNAs participate in molecular regulation by interacting with several proteins [13]. Based on this hypothesis, we identified circFOXP1-interacting proteins by performing an RNA pull-down assay combined with Liquid Chromatography-Mass Spectrometry (LC-MS) in NOZ cells. The RNA-related proteins were determined using SDS-polyacrylamide gel electrophoresis (SDS/PAGE) and silver staining (Fig. 4a). By using LC-MS and comparing the results with those from antisense circFOXP1 experiments, proteins were identified by LC-MS that specifically associated with circFOXP1 (Additional file 6: Table S2). PTBP1 was further detected due to higher expression in the circFOXP1-sense probe compared with the circFOXP1-antisense sample or no RNA sample, and previous studies have verified that PTBP1 is associated with the Warburg effect in cancer cells through regulation of the PKM1/PKM2 ratio [29]. Western blot analysis with anti-PTBP1 antibody indicated the existence of PTBP1 within the circFOXP1 sense RNA probe pull-down samples in NOZ and SGC-996 cells (Fig. 4b). Meanwhile, a RIP assay with PTBP1 antibody showed that endogenous PTBP1 directly bound to circFOXP1 in NOZ and SGC-996 cells (Fig. 4c). Moreover, we observed that knockdown of endogenous circFOXP1 decreased the protein expression of PTBP1 in NOZ and SGC-996 cells, but upregulated expression of circFOXP1 upregulated the expression of PTBP1 in GBC-SD cells (Fig. 4d). By Immunofluorescence and western blot analysis, we further demonstrated that exogenous expression of circFOXP1 enhanced expression of PTBP1 by increased transportation levels of PTBP1 from the nucleus to the cytoplasm, but knockdown of circFOXP1 also revert PTBP1 back to the nucleus (Fig. 5a-b).

### PTBP1 promotes PKLR mRNA expression in GBC cells

In mammalian cells, pyruvate kinase is encoded by 4 isozymes: M1, M2, liver (PKL), and red blood cell (PKR). While the M1, PKL, and PKR isozymes are described to exhibit tissue-specific expression, the pyruvate kinase M2 isoform is highly expressed across cancer types. Glycolytic deregulation, including activation of upregulated PKM2 and liver and RBC (PKLR), is a driver that promotes cancer progression [15, 30, 31]. Previous reports confirmed that PTBP1 regulates AS of the pyruvate kinase gene (PKM) in the acquisition of oncogenic features by increasing recruitment to the PKM pre-mRNA to promote PKM2 splicing, which contributes to the Warburg effect [32]. First, we speculated whether decreased circFOXP1 led to exertion of the function of PTBP1 to regulate AS. The results confirmed that knockdown of endogenous circFOXP1 resulted in no significant change in the mRNA and protein levels of PKM1 in NOZ and SGC-996 cells but had a partial effect on the PKM2 protein level (Fig. 5c-d). PKLR is also known as a promoter in modulation of the Warburg effect [15]. Interestingly, dramatically decreased PKLR expression levels were observed after downregulation of circFOXP1 in NOZ and SGC-996 cells or in NOZ cells in vivo, but increased expression of PKLR was observed after upregulation of circFOXP1 in GBC-SD cells or in vivo (Fig. 5e-f and Additional file 7: Figure S4A). In addition, we showed that PKLR silencing impaired the glycolysis rate and glycolytic capacity in NOZ and SGC-996 cells, which inhibited the Warburg effect in GBC (Additional file 7: Figure S4B-4F).

**Fig. 6:**
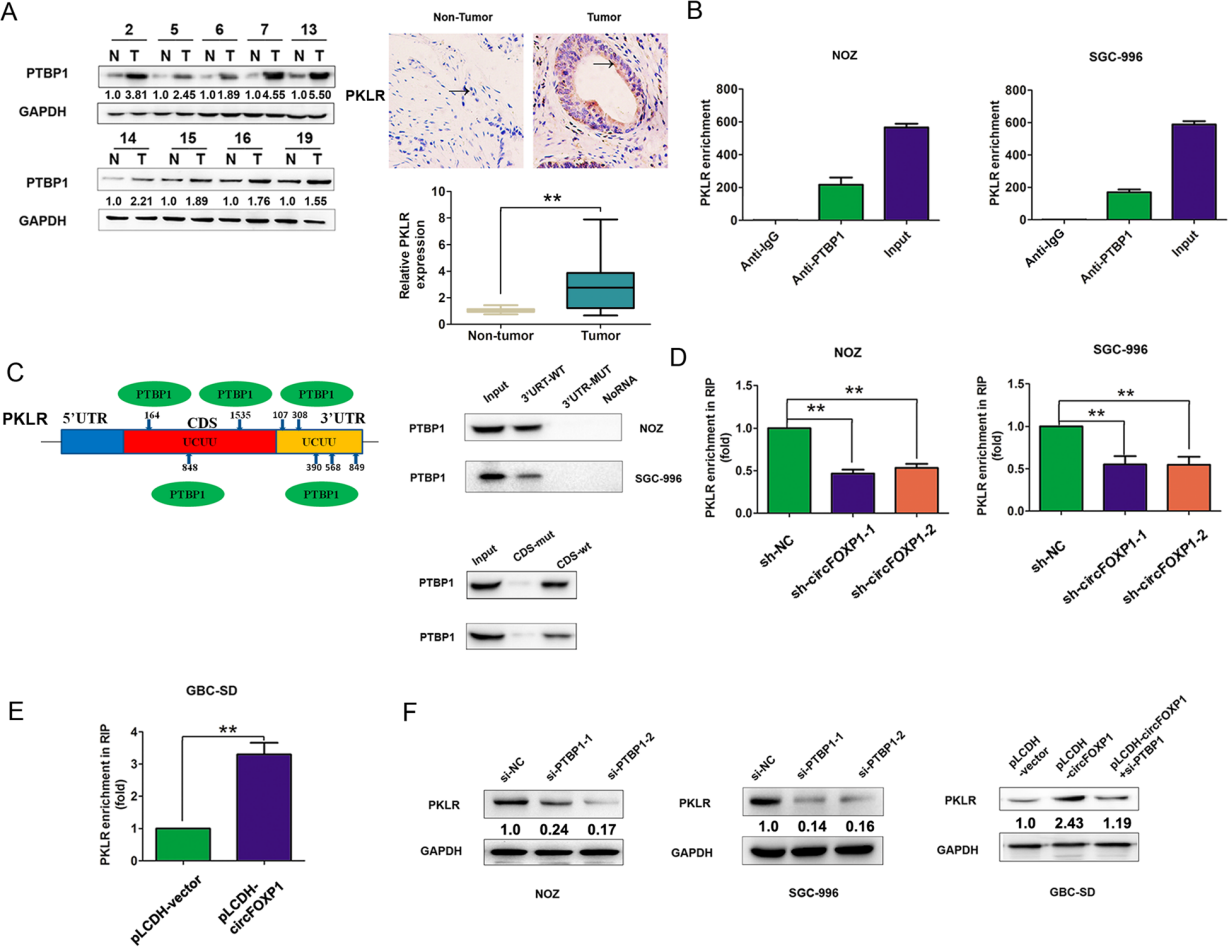
circFOXP1 promotes PKLR expression by interacting with PTBP1 in GBC cells. **a** left, the protein level of PTBP1 was detected using western blot analysis in GBC tissues compared with adjacent normal tissues. Right, immunohistochemical staining and mRNA expression of PKLR in GBC tissues compared with adjacent normal tissues. Arrow, PKLR expression was shown. **b** RIP assays with PTBP1 antibody were performed to detect PKLR enrichment in NOZ and SGC-996 cells. All data are shown as mean ± S.E.M., *n* = 3. **c** A RBP map was used to analyze the binding sites between PTBP1 and the 3’UTR and CDS region of PKLR. PTBP1 was pulled down by the 3’UTR and CDS wt RNA probe but not by the mut RNA probe in NOZ and SGC-996 cells. **d, e** The mRNA enrichment of PKLR was determined using RIP-qRT-PCR after silencing of circFOXP1 in NOZ and SGC-996 cells or overexpression of circFOXP1 in GBC-SD cells. All data are shown as mean ± S.E.M., *n* = 3, **P < 0.01. **f** The expression levels of circFOXP1 were determined using western blot analysis after transfection of NOZ and SGC-996 cells with sh-NC, sh-circFOXP1–1 or sh-circFOXP1–2 or GBC-SD cells with pLCDH-vector, pLCDH-circFOXP1, pLCDH-circFOXP1+ si-PTBP1, *n* = 3 or more

PKLR acts as a driver of tumor growth and metastasis [15, 33]. We sought to elucidate the underlying mechanism by which circFOXP1 affect PKLR expression. PTBP1, an RNA-binding protein, exerts various molecular functions, including RNA metabolism, for example, control of mRNA stability or degradation [34, 35], determination of mRNA localization [36], and protection of mRNAs from decay [37]. Compared with normal tissues, PTBP1 was upregulated in gallbladder cancer tissues and the protein and mRNA of PKLR was also upregulated in gallbladder cancer tissues (Fig. 6a). Furthermore, we showed that the proteasome inhibitor MG-132 could increase PKLR expression and abolished the reduction in PKLR protein levels in circFOXP1-knockdown NOZ cells (Additional file 8: Figure S5A). The protein synthesis inhibitor cycloheximide (CHX) decreased the expression of PKLR proteins by inhibiting protein synthesis, but upregulation of PKLR in circFOXP1-overexpressing SGC-996 and GBC-SD cells was not abolished by treatment with CHX (Additional file 8: Figure S5B). These results showed that circFOXP1 may affect PKLR degradation in GBC.

Based on these findings, we hypothesized that circFOXP1 may enhance the capacity of PTBP1 to bind to PKLR mRNA and protect it from mRNA decay and consequently increasing the PKLR protein levels. To test this hypothesis, we utilized a RIP assay to detect the association between PKLR mRNA and PTBP1. The results demonstrated that PTBP1 could bind to PKLR mRNA in NOZ and SGC-996 cells (Fig. 6b). Using the online prediction software RBP map (http://rbpmap.technion.ac.il/), we found that PTBP1 could bind to the 3’UTR region and coding region (CDS) of PKLR mRNA (UCUU binding bites) (Additional file 9: Figure S6A-6B). Furthermore, an RNA pull-down assay with the 3’UTR region or CDS region of PKLR biotin-labeled RNA probe confirmed that PTBP1 directly bound to PKLR mRNA in NOZ and SGC-996 cells (Fig. 6c). A RIP assay with PTBP1 antibody demonstrated that downregulation of circFOXP1 reduced the enrichment of PKLR mRNA in NOZ and SGC-996 cells, but enhanced the enrichment of PKLR mRNA after overexpression of circFOXP1 in GBC-SD cells (Fig. 6d-e). We further detected the effects of PTBP1 knockdown on PKLR expression. Downregulation of PTBP1 reduced both PKLR mRNA and protein in NOZ and SGC-996 cells, and knockdown of PTBP1 abrogated the effect of overexpressed circFOXP1 on PKLR expression in GBC-SD cells (Additional file 10: Figure S7A and Fig. 6f). These results revealed that circFOXP1 promoted PKLR mRNA expression by interacting with PTBP1 in GBC cells.

### The circFOXP1 binds to miR-370 targeting PKLR in GBC cells

**Fig. 7:**
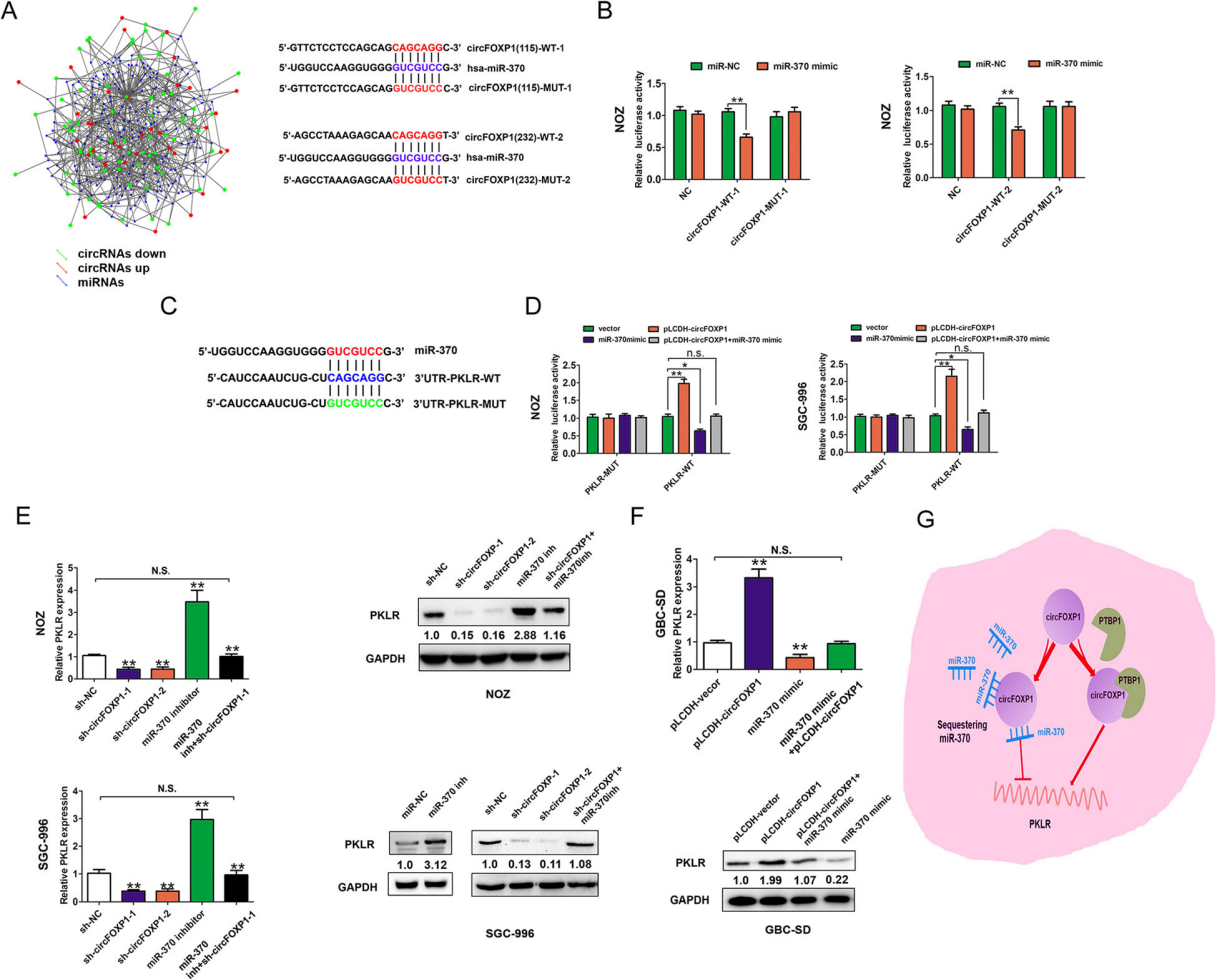
circFOXP1 promotes PKLR expression by binding to miR-370 in GBC cells. **a** MiR-370 have complementary base pairing with circFOXP1 using circRNAs from RNA sequencing targeted miRNAs predicted by miRanda (www.microrna.org) and the online software tools circinteractome (http://circinteractome.nia.nih.gov) (left). The wild type and mutant type complementary sequences of the circFOXP1 and miR-370 binding sequence are shown (right). **b** Luciferase reporter assays were performed in NOZ cells co-transfected with miR-370 mimic or miR-NC and circFOXP1-WT1/2 or circFOXP1-MUT1/2 reporter plasmids. Data are shown as mean ± S.E.M., *n* = 3, **P < 0.01. **c** The wild-type and mutant-type complementary sequences of the PKLR and miR-370 binding sequence are shown. **d** Luciferase reporter assays were performed in NOZ and SGC-996 cells transfected with pLCDH-vector, pLCDH-circFOXP1, miR-370 mimic or co-transfected with miR-370 mimic+pLCDH-circFOXP1 and PKLR-WT or PKLR-MUT reporter vector. Data are shown as mean ± S.E.M., n = 3, *P < 0.05, **P < 0.01, n.s., not significant. **e** The relative expression of PKLR was detected after transfection of NOZ and SGC-996 cells with sh-NC, sh-circFOXP1–1 or sh-circFOXP1–2, miR-370 inhibitor or cotransfection with miR-370 inhibitor and sh-circFOXP1–1. **f** The relative expression of PKLR was detected after transfection of GBC cells with pLCDH-vector, pLCDH-circFOXP1 or cotransfection with pLCDH-circFOXP1 and miR-370 mimic. **g** Proposed mechanism by which circFOXP1 promoted GBC progression by interacting with PTBP1 or sponging miR-370 targeting PKLR. Data are shown as mean ± S.E.M., n = 3 or more, **P < 0.01, n.s., not significant

Endogenous circRNAs have been found to act as microRNA (miRNA) sponges in human cancers [38]. As circFOXP1 was predominantly localized in the cytoplasm, we hypothesized that circFOXP1 could also regulate PKLR expression by binding to specific miRNAs. To confirm this hypothesis, we performed a search for miRNAs that have complementary base pairing with circFOXP1 using the online software tools circinteractome (http://circinteractome.nia.nih.gov) and circRNAs from RNA sequencing targeted miRNAs predicted analysis by miRanda (www.microrna.org) (Fig. 7a, left, Additional file 11: Table S3). The results from RNA sequencing showed that miR-370 could form complementary base pairing with circFOXP1 and PKLR, respectively (Fig. 7a, right, and Fig. 7c). To verify that circFOXP1 could bind to miR-370, the wild-type (WT) and two mutant-type (MUT) circFOXP1 reporter vectors were constructed. We observed that a miR-370 mimic significantly reduced the luciferase activity of the circFOXP1-WT-1 (37%) and circFOXP1-WT-2 (33%) reporter vectors but not that of circFOXP1-MUT-1 or circFOXP1-MUT-2, which suggested that miR-370 was a target of circFOXP1 in a sequence-specific manner (Fig. 7b). To further clarify the regulatory association between circFOXP1 and the 3’UTR of PKLR, we constructed wild-type (WT) and mutant-type (MUT) PKLR 3’UTR reporter vectors (Fig. 7c). The results revealed that the miR-370 mimic significantly reduced the luciferase activity of the WT PKLR 3’UTR reporter vector but not that of the mutant PKLR 3’UTR reporter. Co-transfection with the WT PKLR 3’UTR and miR-370 mimic and pLCDH-circFOXP1 antagonized the effects (Fig. 7d). Furthermore, we confirmed that miR-370 expression was significantly downregulated in GBC tissues and cells, compared with adjacent normal tissues and H69 cell, respectively (Additional file 10: Figure S7B-7C). Moreover, an inverse correlation was observed between circFOXP1 and miR-370 expression in GBC tissues (*r* = − 0.45, *P* < 0.05, Additional file 10: Figure S7D). The PKLR mRNA and protein expression was downregulated after transfection of NOZ and SGC-996 cells with sh-circFOXP1, but was rescued by co-transfection with miR-370 inhibitor and sh-circFOXP1 (Fig. 7e). Conversely, the PKLR mRNA and protein expression was increased after transfection of GBC-SD cells with a pLCDH-circFOXP1, but was rescued by co-transfection with pLCDH-circFOXP1 and miR-370 mimic (Fig. 7f). These results showed that circFOXP1 promoted PKLR expression by sponging miR-370 in GBC cells.

## Discussion

The annotations of circRNAs by transcriptome-wide sequencing in various fundamental cellular biological processes including tumor development and progression have been thrust into the spotlight [39]. Fusion circRNAs (f-circRNAs) produced from transcribed exons of distinct genes were upregulated by cancer-associated chromosomal translocations and subsequently contributed to cellular transformation and enhanced cell viability and resistance upon tumor therapy [40]. In clear cell renal cell carcinoma, AR suppressed circHIAT1 expression by regulating the expression of its host gene (HIAT1) at the transcriptional level, which resulted in deregulation of miR-195-5p/29a-3p/29c-3p, and increased CDC42 to enhance cell migration and invasion [41]. Silencing of circZKSCAN1 promoted cell proliferation, migration and invasion in hepatocellular carcinoma [42]. The circRNA CCDC66 promotes colon cancer growth and metastasis [43]. In spite of some advanced findings, the expression and possible carcinogenic involvement of circRNAs in GBC remains unknown. In this study, we first determined that circFOXP1 was highly upregulated in GBC tissues and cells, and higher circFOXP1 expression was implicated as an independent prognostic marker for OS in patients. Furthermore, upregulation of circFOXP1 expression had pleiotropic effects, including promotion of cell proliferation, migration, invasion, and cell cycle progression and inhibition of cell apoptosis in GBC. These results suggested that circFOXP1 acts as an oncogene and may serve as a prognostic biological marker in GBC.

Mechanistic studies confirmed that circFOXP1 exerted its tumor-promoting roles by modulating the Warburg effect through upregulation of PKLR expression by interacting with PTBP1, protecting PKLR from mRNA decay. PTBP1, an RNA-binding protein, exerts various molecular functions, including RNA metabolism, for example, by repressively regulating AS [44], controlling mRNA stability [34, 35], and determining mRNA localization [36]. The PKM1/M2 isoforms are generated through AS of two mutually exclusive exons, and this AS is controlled by PTBP1 [29]. Herein, we first detected the effects of circFOXP1 on PKM1 and PKM2 expression, and found that decreased circFOXP1 does not significantly change PKM1 expression and only partially affected the PKM2 protein levels, which had been reported in previous studies [32, 45]. Interestingly, we uncovered a novel regulation target of circFOXP1, PKLR, which was activated by upregulation of circFOXP1. We speculated that PTBP1 could affect PKLR mRNA levels by its typical characteristic nucleocytoplasmic shuttling. We demonstrated that circFOXP1 facilitated PTBP1 nuclear to cytoplasmic translocation, resulting in enhancement of the control of PKLR mRNA stability. PTBP1 is critical for post-transcriptional upregulation of proinsulin and other granule proteins biosynthesized shortly after glucose stimulation by stabilizing mRNAs encoding proteins of secretory granules [46, 47]. We found that PTBP1 bound to the 3’UTR and CDS region of PKLR mRNA. Silencing circFOXP1 decreased the ability of PTBP1 to bind PKLR mRNA, which significantly suppressed the Warburg effects in GBC. This is consistent with a previous study that reported PTBP1-mediated post-transcriptional upregulation of proinsulin [46]. Because PTBP1 is mainly located in the nucleus, but circFOXP1 is located in the cytoplasm, we speculated that circFOXP1 could serve as a protein scaffold to recruit not only PTBP1 but also other proteins to the circFOXP1-PTBP1 complex to affect PKLR mRNA expression. Future studies may be necessary to elucidate how circFOXP1 affects the ability of PTBP1 to bind target PKLR mRNA.

Several circRNAs have been found to function as miRNA “sponges” to counteract miRNA-mediated repression of mRNA. CiRS-7, which acts as a designated miR-7 inhibitor, harbors more than 70 conventional miR-7 binding sites and provides a conceptual mechanistic understanding of competing RNA (ceRNA) networks [11]. The circHIPK3 regulates cell growth by binding to miR-124 and inhibiting miR-124 activity [12]. circRNA HRCR functions as an endogenous miR-223 sponge to sequester and inhibit miR-223 activity, which protects the heart from pathological hypertrophy and heart failure [48]. The circPVT1 promotes gastric cancer cell proliferation by acting as a sponge for members of the miR-125 family [49]. Based on the findings, we demonstrated that miR-370 was a direct target of circFOXP1. Upregulation of circFOXP1 promoted PKLR expression by sponging miR-370 in GBC cells.

## Conclusions

Together, our study is the first to reveal that circFOXP1 was upregulated in GBC and promoted the tumor progression in GBC cells by interacting with PTBP1 or sponging miR-370 targeting PKLR (Fig. 7g). These findings have significant implications for our understanding of GBC pathogenesis and provide a target for GBC treatment.

## Supplementary information

**Additional file 1.** Supplementary materials and methods.

**Additional file 2.** The sequences of all primers and oligonucleotide used in the study.

**Additional file 3.** Relative expression levels of several circRNAs in GBC tissues are shown.

**Additional file 4.** CircFOXP1 is identified in GBC cells.

**Additional file 5.** CircFOXP1 promotes cell migration and invasion in GBC.

**Additional file 6.** Proteins are identified by LC-MS that specifically associated with circFOXP1.

**Additional file 7.** Effects of PKLR on Warburg effect in GBC cells.

**Additional file 8.** CircFOXP1 affects PKLR expression in GBC cells.

**Additional file 9.** PTBP1 binds to 3’UTR and CDS region of human PKLR mRNA.

**Additional file 10.** Expression of circFOXP1 is negatively associated with miR-370 in GBC tissues and cells.

**Additional file 11.** CircRNAs from RNA sequencing targeted miRNAs.

## Data Availability

The datasets used and analyzed during the current study are available from the corresponding author on reasonable request.

## Abbreviations

AS: Alternative splicing
CDK2: cyclin-dependent kinase 2
cDNA: complementary DNA
ceRNA: competing endogenous RNA
circFOXP1: circular RNA FOXP1
circRNAs: circular RNAs
FISH: Fluorescence in situ hybridization
GBC: Gallbladder cancer
LC-MS: Liquid Chromatography-Mass Spectrometry
MUT: Mutant-type
ncRNA: non-coding RNA
OS: Overall survival
PCNA: Proliferating cell nuclear antigen
PKLR: pyruvate kinase, liver and RBC
qRT-PCR: quantitative real-time PCR
RIP: RNA immunoprecipitation
SDS/PAGE: SDS-polyacrylamide gel electrophoresis
shRNA: short hairpin RNA
WT: wild-type
