# Supplementary material for: Circular RNA FOXP1 promotes tumor progression and Warburg effect in gallbladder cancer by regulating PKLR expression
Source: Mol Cancer. 2019 Oct 17;18:145. doi: 10.1186/s12943-019-1078-z (PMC6796492; doi:10.1186/s12943-019-1078-z)
Supplement: Supplementary file 1 — Additional file 1. Supplementary materials and methods. [file 12943_2019_1078_MOESM1_ESM.doc]

**Supplementary Materials and Methods**

**RNA-seq analysis**

Total RNA from four GBC tissues and paired adjacent normal tissues was isolated using Hipure Total RNA Mini Kit (Magen) according to the protocol. RNAs were eluted with 50μl of RNase-free water and then re-purified the concentration and integrity of the extracted total RNA was estimated by Qubit 3.0 Fluorometer (Invitrogen, Carlsbad, California), and Agilent 2100 Bioanalyzer (Applied Biosystems, Carlsbad, CA), respectively. RNA samples with a RIN value of at least 7.0 or higher was used for further processing. RNA-seq library was prepared with approximately 1μg of total RNA using KAPA Stranded RNA-Seq Kit with RiboErase (HMR) for Illumina Platforms (Kapa Biosystems, Inc., Woburn, MA). Briefly, ribosomal RNA was removed from total RNA. Next, first strand and directional second strand synthesis were performed. Then the A tailing and adapter ligation were performed with the purified cDNA. Finally, the purified, adapter-ligated DNA was amplified. The library quality and concentration was assessed by utilizing a DNA 1000 chip on an Agilent 2100 Bioanalyzer. Accurate quantification for sequencing applications was determined using the qPCR-based KAPA Biosystems Library Quantification kit (Kapa Biosystems, Inc., Woburn, MA). Each library was diluted to a final concentration of 10 nM and pooled equimolar prior to clustering. Paired-End (PE) sequencing was performed on all samples. For circRNA expression analysis, the read was to mapped genome using the STAR and DCC was used to identify the cirRNAs and to estimate the circRNAs expression. TMM (trimmed mean of M-values) was used to normalize the gene expression. Differentially expressed genes were identified using the edgeR program.

**Cell transfection and vector construction**

The shRNA sequences against circFOXP1 were cloned into a pGPU6/GFP/Neo vector (GenePharma Co., Ltd, Shanghai, China). The pLCDH-circFOXP1 was synthesized using full-length circFOXP1 (hsa_circ_0008234) and subcloned into a pLCDH-vector with the cloning sites BamHI/EcoRI (GENESEED, Guangzhou, China). WT circFOXP1 with potential miR-370 binding sites and circFOXP1 with mutation of two sites or WT/MUT PKLR-3’UTR with potential miR-370 binding sites were constructed by chemosynthesis (Sangon Biotech, China) and fused to the luciferase reporter vector psiCHECK2 (Promega, Madison, WI, USA). Cells were transfected using Lipofectamine TM 3000 (Invitrogen) according to the manufacturer’s instructions.

**Cell Counting Kit-8 (CCK-8) assay**

Cell proliferation capacity was evaluated using Cell Counting Kit-8 (CCK-8) (Dojindo, Japan). Briefly, 2000 GBC cells/well were seeded into 96-well plates and then transfected with sh-NC, sh-circFOXP1-1 and sh-circFOXP1-2 or pLCDH-vector and pLCDH-circFOXP1. At 0, 1, 2, 3, 4, 5, 6 and 7 days after cell transfection, 10 μl of CCK-8 assay solution was added to each well. Then, after incubation for another 2 h, the optical density at 450 nm was measured with an enzyme immunoassay analyzer (Thermo Fisher Scientific, Inc., Waltham, MA, USA).

**Cell migration and invasion assays**

Cell migration and invasion assays were performed in Transwell chambers (8-μm pore size, Corning Costar Corp., Corning, NY, USA) precoated with (for invasion assay) or without (for migration assay) Matrigel (BD, NJ, USA). Cells were harvested 48 h after transfection and then 1× 10^5^ cells incubated with serum-free DMEM were added to the upper chamber, and DMEM with 10% FBS was added to the lower chamber. The chambers were then incubated for 36 h before examination. The cells on the upper surface were removed, while the cells on the lower surface were fixed with methanol for 20 min and stained with 1% crystal violet. Finally, cells were counted in 10 random fields under a microscope (Olympus, Shanghai, China).

**RNA extraction and qRT-PCR analysis**

Total RNA derived from GBC tissues and cells was extracted using TRIzol reagent (TAKARA, Dalian, China) according to the manufacturer’s instructions. RNA was reverse transcribed into cDNA using a Primer-Script one step RT-PCR kit (TAKARA, Dalian, China). Quantitative real-time PCR experiments were performed using a SYBR Premix Dimmer Eraser kit (TAKARA, Dalian, China) on an ABI 7500 Real-Time PCR System (Applied Biosystems). Relative mRNA or mircoRNA expression was normalized to GADPH or U6 expression. The primer sequences used are shown in Supplementary Table 2. The fold change in relative expression of mRNAs was calculated using the 2^−ΔΔCt^ methods.

**Western blot analysis**

Western blot was performed as described previously . Monoclonal or polyclonal antibodies against PCNA (Abcam, USA), PKM1 (Proteintech Group, USA), PKM2 (Proteintech Group, USA), PKLR (Cell Signaling Technology, USA), PTBP1 (Abcam, USA), AKT (Cell Signaling Technology, USA), Caspase-3 (Cell Signaling Technology, USA), MMP9 (Cell Signaling Technology, USA) and Ki-67 (Cell Signaling Technology, USA) were used in the study. GAPDH served as a loading control (Cell Signaling Technology, USA). Blots were incubated with anti-rabbit secondary antibody (1:3000; Cell Signaling Technology, Shanghai) and visualized using enhanced chemiluminescence (Thermo Scientific, Shanghai, China). All experiments were performed in triplicate.

**Flow cytometric analysis**

After 48 h of incubation, transfected GBC cells were washed with cold phosphate-buffered saline (PBS) and incubated in ice-cold 70% ethanol at 4 °C overnight. Then, cells were incubated with propidium iodide for 30 min and analyzed for cell cycle distribution using a flow cytometer (FACS Calibur, BD Biosciences, USA). The data are presented as the percentage of cell phase distribution including G0/G1, S and G2/M phases. For cell apoptosis analysis, the cultured cells were stained using annexin V-fluorescein isothiocyanate (Beyotime, Nantong, China), and the apoptosis rates were measured using a flow cytometer (FACSCalibur; BD Biosciences, Sparks, MD, USA).

**Immunohistochemistry (IHC) and immunofluorescence assays**

IHC was performed as described previously . Tumor tissues were fixed using 4% paraformaldehyde and embedded in paraffin. The samples were then incubated with primary antibody against Ki-67, treated with secondary antibody for 30 min and stained with diaminobenzidine (DAB) until brown granules appeared. For immunofluorescence assays, transfected cells were fixed with 4% paraformaldehyde and incubated with primary antibody with PTBP1 (Proteintech Group, USA) at 4 °C overnight and then incubated with goat anti-rabbit IgG with a red fluorescent label (Invitrogen, Carlsbad, CA).

**Luciferase reporter assay**

To demonstrate the circFOXP1 was a target of miR-370, in brief, 5×10^3^ GBC cells seeded in a 96-well plate were co-transfected with 150 ng of empty psicheck2 vector, psicheck2-circFOXP1-WT1/2 or psicheck2-circFOXP1-MUT1/2 (Sangon Biotech, Shanghai, China) and 2ng of pRL-TK (Promega, Madison, WI, USA) with a miR-370 mimic or miR-NC using Lipofectamine 2000 (Invitrogen, Carlsbad, California, USA). To demonstrated the PKLR was a target of circFOXP1, 5×10^3^ GBC cells seeded in a 96-well plate were co-transfected with 150 ng of vector, pLCDH-cirFOXP1, miR-370 mimic or pLCDH+ miR-370 mimic and psicheck2-PKLR-3’UTR-WT/MUT (Sangon Biotech, Shanghai, China) and 2ng of pRL-TK (Promega, Madison, WI, USA) using Lipofectamine 2000 (Invitrogen, Carlsbad, California, USA). After cell transfection for 48 h, the luciferase activity in cell lysates was analyzed using a Dual-Luciferase Reporter Assay System (Promega) according to the manufacturer's instructions. The relative luciferase activity was normalized to Renilla luciferase activity.

**Northern blot assay**

Northern blotting was performed using a Northern Max Kit from Ambion (Life Technologies) as described previously with minor modifications . Digoxin-labeled RNA probes were prepared with a DIG Northern starter Kit (Roche, Indianapolis, IN, USA) with the corresponding PCR products as templates for T7 transcription. The PCR primers were circFOXP1-F,5’-TCTGACCACGACATGTGTCT-3’;andcircFOXP1-R,5’-CTTGAAGCTGCTGCTGTTGAA-3’. Then, 10 μg of total RNA was run on a 2% agarose gel and transferred to a Hybond-N+membrane (GE Healthcare, Uppsala, Sweden) by capillary transfer. Membranes were dried and cross-linked via exposure to ultraviolet (265 nm) light 1×at 200,000 μ J cm^−2^. Pre-hybridization was conducted at 62 °C for 1 h, and hybridization was performed at 62 °C overnight. The membrane was washed twice with 5× saline-sodium citrate (SSC), 1% SDS, once with 1× SSC, 1% SDS and wrapped in saran wrap. Signals were detected by exposure to a screen and scanning with a PMI (Bio-Rad).

**Fluorescence in situ hybridization (FISH)**

RNA FISH was performed as previously described elsewhere with minor modification . A biotin-labeled RNA probe (5’-AGCCACTGACACGGGAACCTTTCCCTTTTGGGAGT-3’) was constructed by Goodbio (Shanghai, China). NOZ and SGC-996 cells were cultured to the exponential phase and were approximately 90% confluent at the time of fixation. Then, cells were fixed using 4% formaldehyde for 25 min and washed 3 times in PBS/PVA for 5 min. Cells were permeabilized with 1% Triton X-100 in PBS for 30 min and then washed in 2× SSC plus 50% formamide for 5 min. Cells were hybridized in hybridization buffer (40% formamide, 10% dextran sulfate, 1×Denhardt's solution, 4×SSC, 10 mM DDT, 1 mg ml−1 yeast transfer RNA, 1 mg ml−1 sheared salmon sperm DNA) and 4 ng/µl of RNA probe in 2×SSC plus 50% formamide 60 °C overnight. Signals were detected using a tyramide-conjugated Alexa 488 fluorochrome TSA kit (Life Technologies).

**Extracellular acidification rate/oxygen consumption rate measurements**

The extracellular acidification rate (ECAR) was measured using a Seahorse XF24 analyzer (Seahorse Biosciences) as described previously . Briefly, after cell transfection, 1×10^4^ cells/well was cultured in a 96-well XF microplate (Seahorse Biosciences) overnight. Cells were washed before incubation with assay medium (0.5 mL) for 1 h at 37 °C. Then, cartridge ports A, B, and C were loaded with 75 μL of glucose (80 mM), oligomycin (9 μM), and 2-DG (1 M), respectively. The ECAR after glucose treatment indicated the glycolysis rate. The ECAR after oligomycin treatment indicated the glycolysis capacity. After the probes were calibrated, for OCR, 1mM oligomycin, 1mM p-trifluoromethoxy carbonyl cyanide phenylhydrazone and 2mM antimycin A plus 2mM rotenone (Rote/AA) were sequentially injected, Data were assessed and analyzed by using Seahorse XF24 analyzer software.

**Supplementary legends**

**Supplementary Fig. 1 Relative expression levels of several circRNAs in GBC tissues were shown.** (A) Relative expression levels of circXPO1, circMAPK1, circSENP1, and circSMAD2 were evaluated in human GBC tissues and adjacent normal tissues (n=40). The mRNA expression was normalized to that of GADPH. (B) Association between circSMAD2 or circMAPK1 and OS of GBC patients was shown using Kaplan-Meier analysis and log rank tests.

**Supplementary Fig. 2** **CircFOXP1 is identified in GBC cells.** (A) CircFOXP1 expression levels were evaluated in human GBC cell lines (NOZ, GBC-SD, EHGB-1, SGC-996 and OCUG-1) and a human intrahepatic biliary epithelial cell line (H69), another normal biliary epithelia cell line HIBEC using qRT-PCR. The expression of circFOXP1 was normalized to that of GADPH. Significant differences between groups were analyzed with a paired samples t-test, *P<0.05, **P<0.01. (B) The corresponding exons were labeled and arrowheads indicated the backsplice junctions. (C) QRT-PCR was performed to detect the circFOXP1 from control RNA, circHIPK3 or digested RNAs using RNase R exonuclease in five GBC cell lines. (D) The relative mRNA expression of circFOXP1 in nucleus or cytoplasm in NOZ or SGC-996 cells was determined by qRT-PCR. GAPDH was used as cytoplasm control and U1 was used as nuclear control. (E)-(F) The relative mRNA expression of circFOXP1 and FOXP1 after transfected with sh-NC, sh-circFOXP1-1 or sh-circFOXP1-2 in NOZ and SGC-996 cells or transfected with pLCDH-vector or pLCDH-circFOXP1, pLCDH-circFOXP1+RNase R in GBC-SD and EHGB-1 cells were determined by qRT-PCR, Data are shown as mean± S.E.M., n=3 or more, **P<0.01.

**Supplementary Fig. 3 CircFOXP1 promotes cell migration and invasion in GBC.** (A) The expression of Proliferating Cell Nuclear Antigen (PCNA), Caspase-3, MMP9 and AKT was detected after transfection of NOZ and SGC-996 cells with sh-NC, sh-circFOXP1-1 or sh-circFOXP1-2 or GBC-SD cells with pLCDH-vector and pLCDH-circFOXP1 by western blot analysis (n=3). (B) The cell migration and invasion capacities were assessed after transfected with sh-NC, sh-circFOXP1-1 or sh-circFOXP1-2 in NOZ and SGC-996 cells. (C) The cell migration and invasion capacities were assessed after transfected with pLCDH-vector or pLCDH-circFOXP1 in GBC-SD cells. Statistical analysis was conducted using student *t*-test analysis. Data are shown as mean± S.E.M., n=3, **P<0.01, original magnification, 200×.

**Supplementary Fig. 4 Effects of PKLR on Warburg effect in GBC cells.** (A) The relative PKLR expression level was detected by western blot analysis in vivo, mice were implanted with GBC-SD cells (top) transfected with pLCDH-vector or pLCDH-circFOXP1 and NOZ cells (low) transfected with lv-sh-NC, lv-sh-circFOXP1-1 or lv-sh-circFOXP1-2. (B) The relative mRNA expression of PKLR was detected by qRT-PCR after cells were transfected with si-NC or si-PKLR in NOZ or SGC-996 cells. (C)-(F) The extracellular acidification rate was analyzed after transfection of NOZ or SGC-996 cells with si-NC and si-PKLR. The extracellular acidification rate after glucose treatment indicates the glycolysis rate. The extracellular acidification rate after oligomycin treatment indicates the glycolysis capacity. Abbreviations: 2‐DG, 2-deoxy-d-glucose; ECAR, extracellular acidification rate. **P<0.01.

**Supplementary Fig. 5 CircFOXP1 affects PKLR expression in GBC cells.** (A) The control cells (top) and circFOXP1 stable knockdown NOZ (low) cells were incubated with MG132 (5μmol/L) for 24 hours. The PKLR protein expression were detected by Western blots (n = 3). (B) The control cells (top) and circFOXP1 stable overexpressed SGC-996 or GBC-SD cells (low) were incubated with the protein synthesis inhibitor CHX (0.5 μg/μL) for 24 hours, The PKLR protein expression were detected by Western blots (n = 3).

**Supplementary Fig. 6 PTBP1 binds to 3’UTR and CDS region of human PKLR mRNA.** (a) The sites of PTBP1 binding to 3’UTR were shown. (b) The sites of PTBP1 binding to CDS region were shown.

**Supplementary Fig. 7 Expression of circFOXP1 is negatively associated with miR-370 in GBC tissues and cells.** (A) The relative mRNA expression of PTBP1 was detected by qRT-PCR after cells was transfected with si-NC, si-PTBP1-1 and si-PTBP1-2 in NOZ or SGC-996 cells. (B) The relative expression of miR-370 in tissues from 40 cases of GBC compared with adjacent normal tissues was detected by qRT-PCR. The expression of miR-370 was normalized to U6. Significant differences between groups were analyzed with a paired samples *t-*test, ***P<0.001. (C) The relative expression of miR-370 in GBC cells and H69 cell was detected by qRT-PCR. The expression of miR-370 was normalized to U6. (D) The association between circFOXP1 and miR-370 expression in GBC tissues was determined by Pearson correlation coefficient analysis (r=-.0.450, P<0.05).

**References**

1. Dobin A, Davis CA, Schlesinger F, Drenkow J, Zaleski C, Jha S, Batut P, Chaisson M, Gingeras TR: **STAR: ultrafast universal RNA-seq aligner.** *Bioinformatics* 2013, **29:**15-21.

2. Cheng J, Metge F, Dieterich C: **Specific identification and quantification of circular RNAs from sequencing data.** *Bioinformatics* 2016, **32:**1094-1096.

3. Wang SH, Ma F, Tang ZH, Wu XC, Cai Q, Zhang MD, Weng MZ, Zhou D, Wang JD, Quan ZW: **Long non-coding RNA H19 regulates FOXM1 expression by competitively binding endogenous miR-342-3p in gallbladder cancer.** *J Exp Clin Cancer Res* 2016, **35:**160.

4. Huang C, Cao L, Qiu L, Dai X, Ma L, Zhou Y, Li H, Gao M, Li W, Zhang Q, et al: **Upregulation of H19 promotes invasion and induces epithelial-to-mesenchymal transition in esophageal cancer.** *Oncol Lett* 2015, **10:**291-296.

5. Wang SH, Wu XC, Zhang MD, Weng MZ, Zhou D, Quan ZW: **Upregulation of H19 indicates a poor prognosis in gallbladder carcinoma and promotes epithelial-mesenchymal transition.** *Am J Cancer Res* 2016, **6:**15-26.

6. Dueck A, Ziegler C, Eichner A, Berezikov E, Meister G: **microRNAs associated with the different human Argonaute proteins.** *Nucleic Acids Res* 2012, **40:**9850-9862.

7. Zheng Q, Bao C, Guo W, Li S, Chen J, Chen B, Luo Y, Lyu D, Li Y, Shi G, et al: **Circular RNA profiling reveals an abundant circHIPK3 that regulates cell growth by sponging multiple miRNAs.** *Nat Commun* 2016, **7:**11215.

8. Nie H, Li J, Yang XM, Cao QZ, Feng MX, Xue F, Wei L, Qin W, Gu J, Xia Q, Zhang ZG: **Mineralocorticoid receptor suppresses cancer progression and the Warburg effect by modulating the miR-338-3p-PKLR axis in hepatocellular carcinoma.** *Hepatology* 2015, **62:**1145-1159.
