# Supplementary material for: Circular RNA FOXP1 promotes tumor progression and Warburg effect in gallbladder cancer by regulating PKLR expression
Source: Mol Cancer. 2019 Oct 17;18:145. doi: 10.1186/s12943-019-1078-z (PMC6796492; doi:10.1186/s12943-019-1078-z)
Supplement: Supplementary file 2 — Additional file 2. The sequences of all primers and oligonucleotide used in the study. [file 12943_2019_1078_MOESM2_ESM.doc]

**Table 2** The sequences of all primers and oligonucleotide used in the study.

| Gene | Forward primer (5’ to 3’) | Reverse primer (5’ to 3’) |
| --- | --- | --- |
| GAPDH | CGGAGTCAACGGATTTGGTCGTAT | AGCCTTCTCCATGGTGGTGAAGAC |
| U6 | CTCGCTTCGGCAGCACA | AACGCTTCACGAATTTGCGT |
| circFOXP1 | CCACATGCCTCTACCAATGGA | CAGCACTTGTTGCTGGAGGAT |
| circXPO1 | TTCACATACTGCTGGTTCAT | TGCCTGCTGGATTATACTATC |
| circMAPK1 | GGTTCCTGACAGAATATGTG | AGTAGGTCTGGTGCTCAA |
| circSMAD2 | CAGGAATTGAGCCACAGA | CCAGCAGTCTCTTCACAA |
| circSENP1 | AACCTGTTCCAAGTCCATC | AGCATCCATCCTCATCCTA |
| PKM1 | CGAGCCTCAAGTCACTCCAC | GTGAGCAGACCTGCCAGACT |
| PKM2 | ATTATTTGAGGAACTCCGCCGCCT | ATTCCGGGTCACAGCAATGATGG |
| PKLR | TGGGAAAACTGGGTGGGATGGATG | GAAGGAAGCAGCCGGGGATTTGAC |
| miR-370 | GCCTGCTGGGGTGGAACCTGGTAA |  |
| RIP qRT-PCR | CTCTGCACCTTCCAAGACCT | TGCATTTGCTGGGGAGTGATA |
| Oligonucleotide | Sense (5’ to 3’) | Antisense (5’ to 3’) |
| sh-circFOXP1-1 | CACCGAAAGGGAAAGGTTCCCGTGTCTTCAAGAGAGACACGGGAACCTTTCCCTTTTTTTTTG | GATCCAAAAAAAAAGGGAAAGGTTCCCGTGTCTCTCTTGAAGACACGGGAACCTTTCCCTTTC |
| sh-circFOXP1-2 | CACCGCTCCCAAAAGGGAAAGGTTCCTTCAAGAGAGGAACCTTTCCCTTTTGGGAGTTTTTTG | GATCCAAAAAACTCCCAAAAGGGAAAGGTTCCTCTCTTGAAGGAACCTTTCCCTTTTGGGAGC |
| si-NC | UUCUCCGAACGUGUCACGUTT | ACGUGACACGUUCGGAGAATT |
| si-PTBP1-1 | GCCUCAACGUCAAGUACAAdTdT | UUGUACUUGACGUUGAGGCdTdT |
| si-PTBP1-2 | GCGUCGUCAAAGGAUUCAAdTdT | UUGAAUCCUUUGACGACGCdTdT |
| si-PKLR | GCAGCUGUUUGAGGAGCUATT | UAGCUCCUCAAACAGCUGCTT |
