# Supplementary figures and images for: Circular RNA FOXP1 promotes tumor progression and Warburg effect in gallbladder cancer by regulating PKLR expression
Source: Mol Cancer. 2019 Oct 17;18:145. doi: 10.1186/s12943-019-1078-z (PMC6796492; doi:10.1186/s12943-019-1078-z)

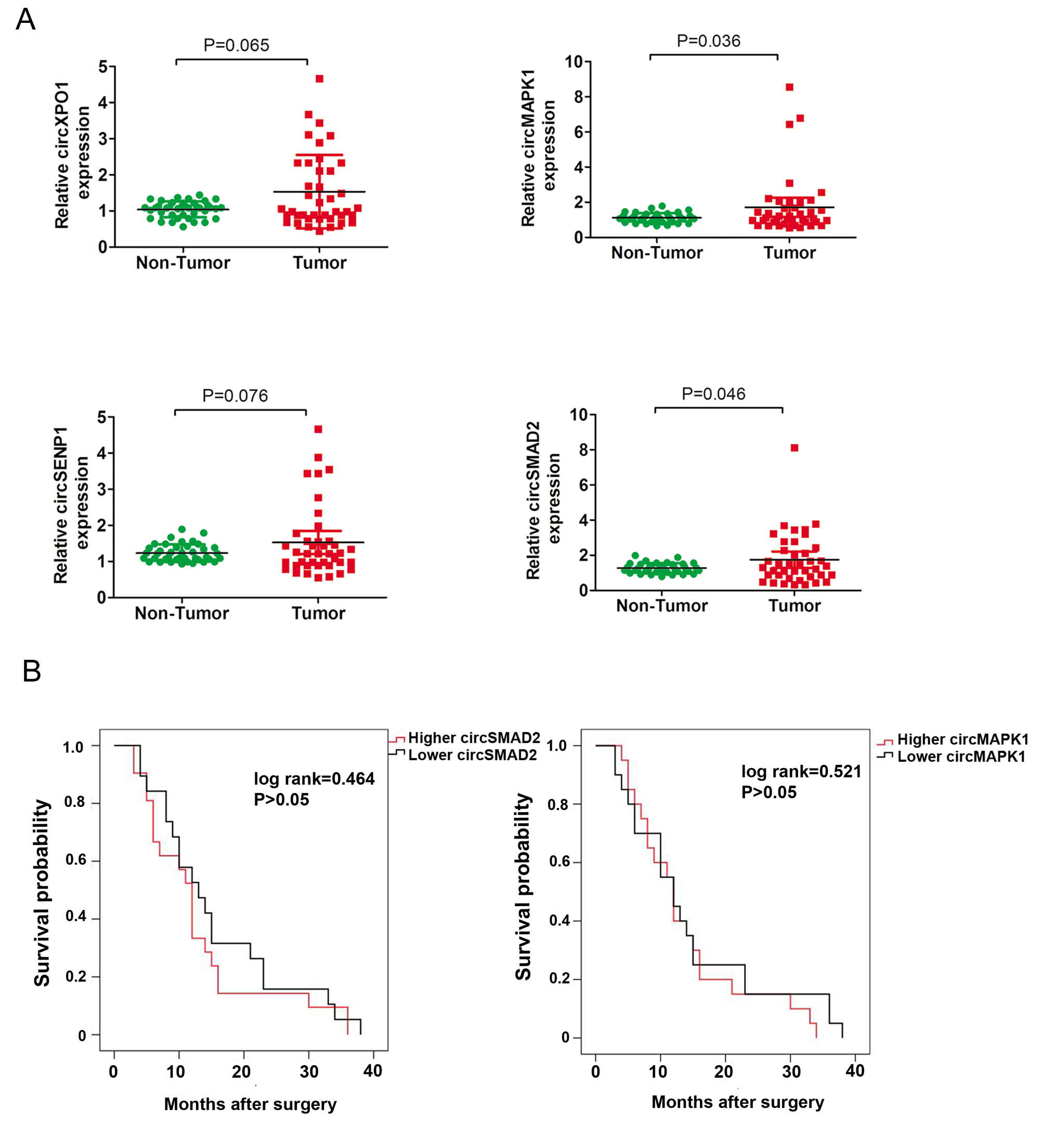

Supplement: Supplementary file 3 — Additional file 3. Relative expression levels of several circRNAs in GBC tissues are shown. [file 12943_2019_1078_MOESM3_ESM.tif]

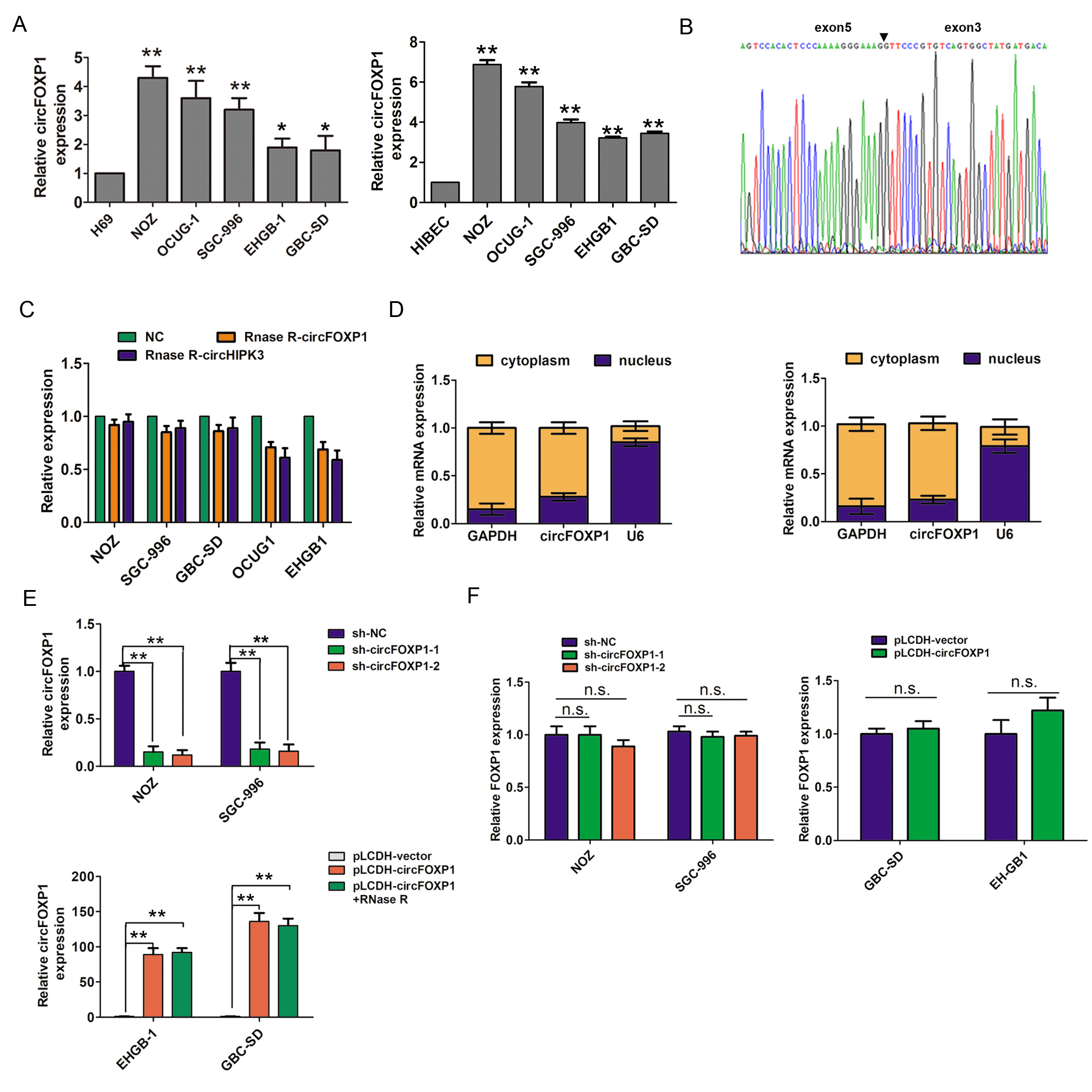

Supplement: Supplementary file 4 — Additional file 4. CircFOXP1 is identified in GBC cells. [file 12943_2019_1078_MOESM4_ESM.tif]

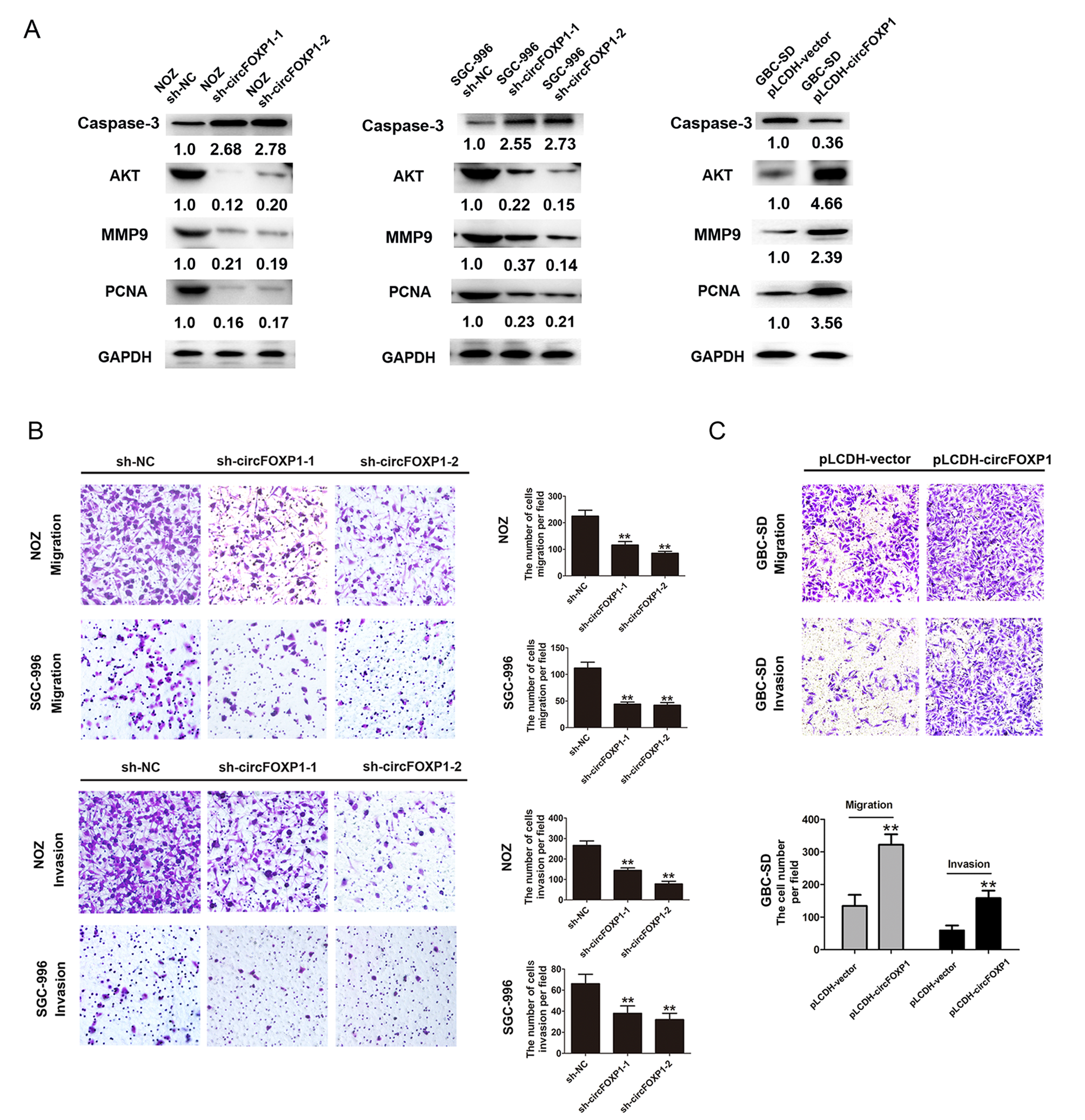

Supplement: Supplementary file 5 — Additional file 5. CircFOXP1 promotes cell migration and invasion in GBC. [file 12943_2019_1078_MOESM5_ESM.tif]

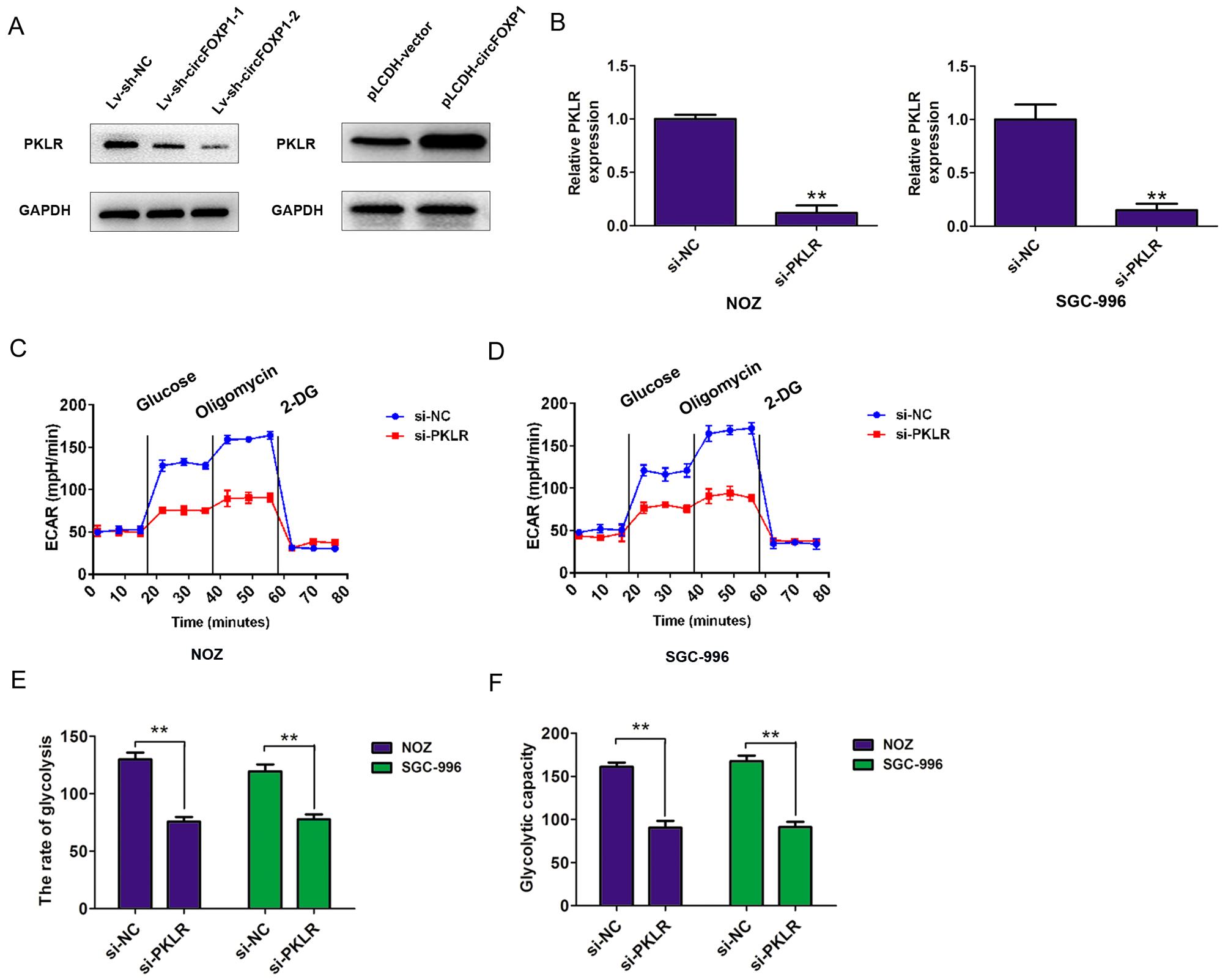

Supplement: Supplementary file 7 — Additional file 7. Effects of PKLR on Warburg effect in GBC cells. [file 12943_2019_1078_MOESM7_ESM.tif]

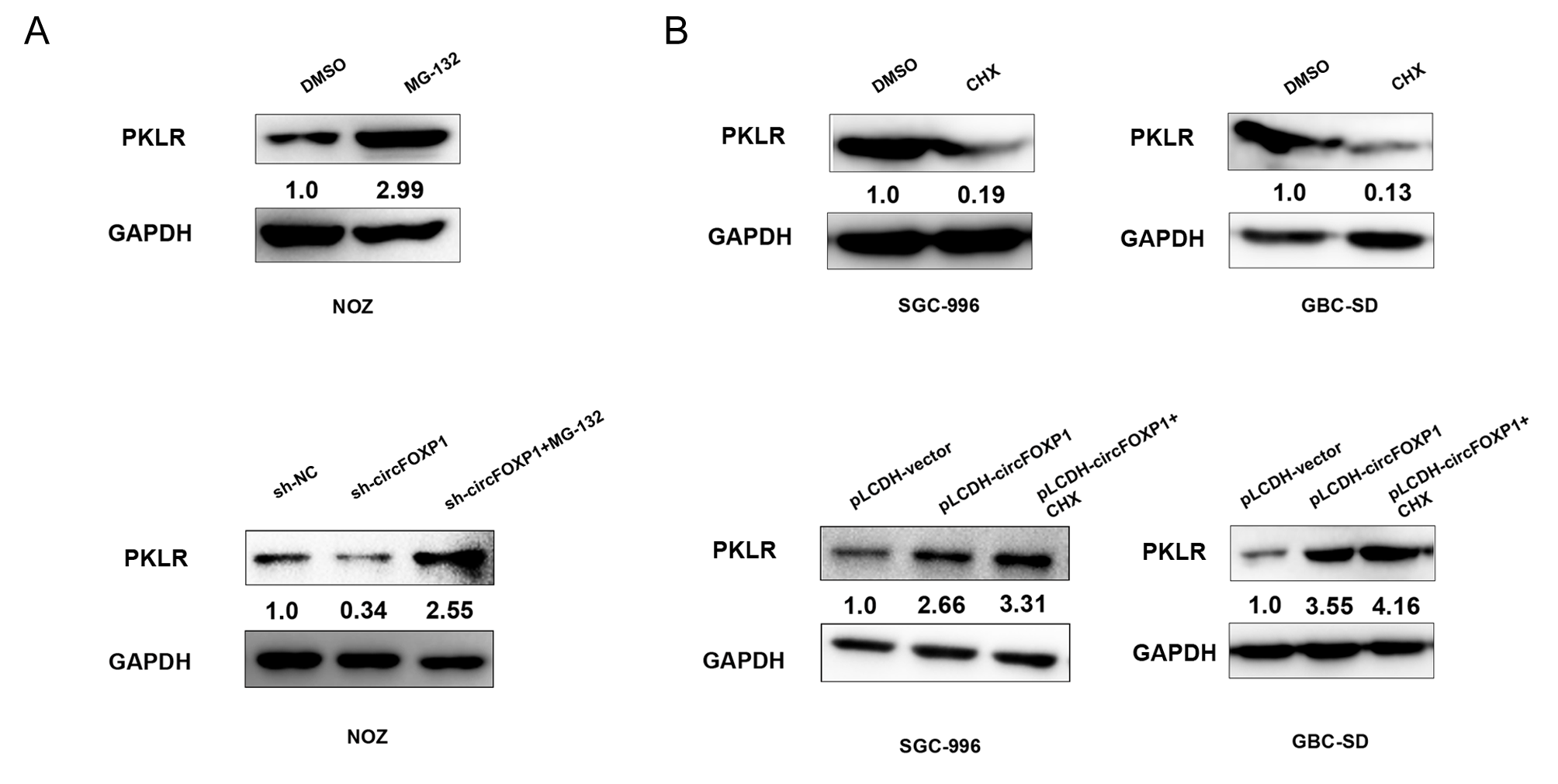

Supplement: Supplementary file 8 — Additional file 8. CircFOXP1 affects PKLR expression in GBC cells. [file 12943_2019_1078_MOESM8_ESM.tif]

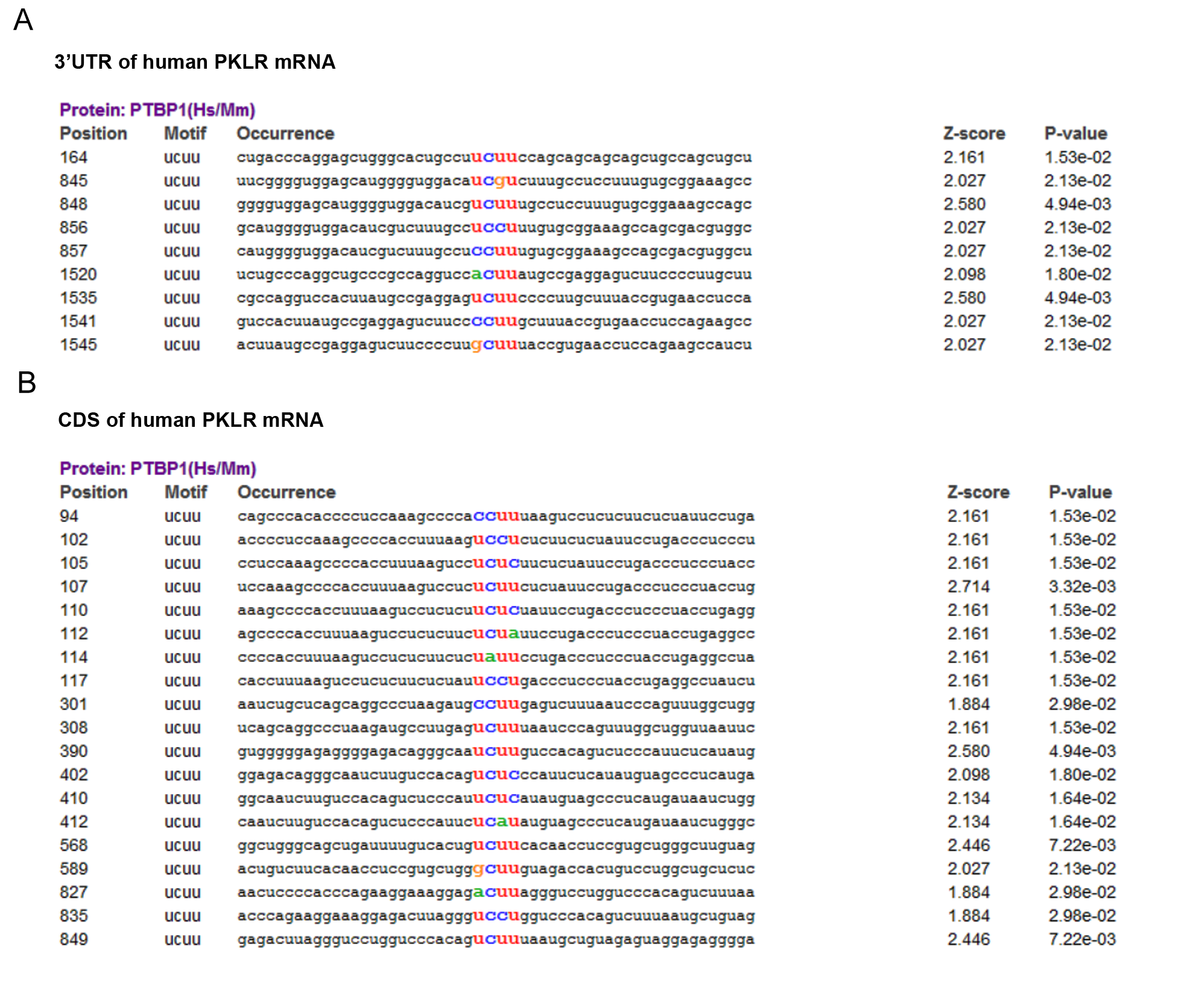

Supplement: Supplementary file 9 — Additional file 9. PTBP1 binds to 3’UTR and CDS region of human PKLR mRNA. [file 12943_2019_1078_MOESM9_ESM.tif]

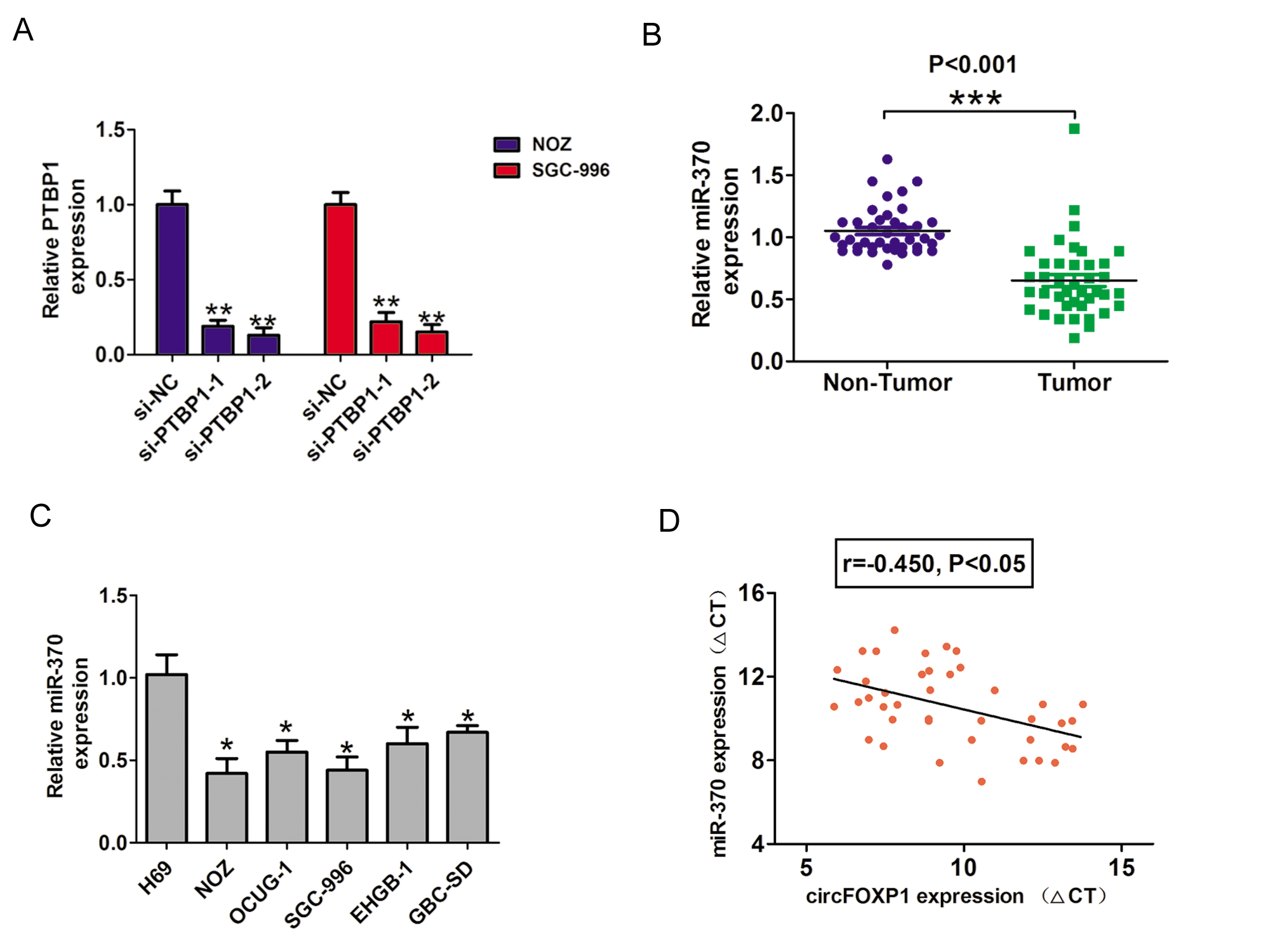

Supplement: Supplementary file 10 — Additional file 10. Expression of circFOXP1 is negatively associated with miR-370 in GBC tissues and cells. [file 12943_2019_1078_MOESM10_ESM.tif]
